# Efficacy and mechanisms of traditional Chinese medicine for COVID-19: a systematic review

**DOI:** 10.1186/s13020-022-00587-7

**Published:** 2022-02-28

**Authors:** Xiaomin Kang, De Jin, Linlin Jiang, Yuqing Zhang, Yuehong Zhang, Xuedong An, Liyun Duan, Cunqing Yang, Rongrong Zhou, Yingying Duan, Yuting Sun, Fengmei Lian

**Affiliations:** 1 Guang’anmen Hospital, China Academy of Chinese Medical Sciences, Beijing, China; 2 Beijing University of Chinese Medicine, Beijing, China

**Keywords:** Traditional Chinese medicine, Coronavirus disease 2019, COVID-19, Efficacy, Mechanism, Systematic review

## Abstract

Since the outbreak of coronavirus disease 2019 (COVID-19), traditional Chinese medicine (TCM) has made an important contribution to the prevention and control of the epidemic. This review aimed to evaluate the efficacy and explore the mechanisms of TCM for COVID-19. We systematically searched 7 databases from their inception up to July 21, 2021, to distinguish randomized controlled trials (RCTs), cohort studies (CSs), and case–control studies (CCSs) of TCM for COVID-19. Two reviewers independently completed the screening of literature, extraction of data, and quality assessment of included studies. Meta-analysis was performed using Review Manager 5.4 software. Eventually, 29 RCTs involving 3060 patients and 28 retrospective studies (RSs) involving 12,460 patients were included. The meta-analysis demonstrated that TCM could decrease the proportion of patients progressing to severe cases by 55% and the mortality rate of severe or critical patients by 49%. Moreover, TCM could relieve clinical symptoms, curtail the length of hospital stay, improve laboratory indicators, and so on. In addition, we consulted the literature and obtained 149 components of Chinese medicinal herbs that could stably bind to antiviral targets or anti-inflammatory or immune-regulating targets by the prediction of molecular docking. It suggested that the mechanisms involved anti-virus, anti-inflammation, and regulation of immunity. Our study made a systematic review on the efficacy of TCM for COVID-19 and discussed the possible mechanisms, which provided clinical reference and theoretical basis for further research on the mechanism of TCM for COVID-19.

**Supplementary Information:**

The online version contains supplementary material available at 10.1186/s13020-022-00587-7.

## Introduction

Coronavirus disease 2019 (COVID-19) is a globally widespread acute respiratory infection due to infection with 2019 novel coronavirus (2019n-CoV, also named SARS-CoV-2), which continues to threaten human health and development. However, China has managed to get the situation under control through various measures, specifically, traditional Chinese medicine (TCM) performed a significant and indispensable function in fighting the epidemic [1]. Previous studies have shown that TCM could suppress virus entry, replication, and transcription, and reduce the immune disorders and cytokine storm caused by viral infection [2]. Abundant clinical evidence supports that TCM has a significant improvement effect on COVID-19, such as improving lung CT, shortening the conversion time of negative results of the 2019-nCoV nucleic acids tests, alleviating clinical symptoms, and promoting recovery [3]. Previous systematic reviews that evaluated the effectiveness and safety of TCM for COVID-19 did not systematically appraise the proportion of patients progressing to severe cases and the mortality rate of severe or critical patients, and more importantly, did not divide subgroups based on clinical types [4–6]. The prognosis of COVID-19 patients correlates greatly with the clinical types, and it is not appropriate to put patients with different clinical types together for evaluation. Therefore, this study evaluated the efficacy of TCM for COVID-19 after grouping patients according to their different clinical types.

## Methods

We performed this study in accordance with the PRISMA 2020 statement [7]. The protocol for this review has been registered in PROSPERO (CRD42021269173).

### Criteria for considering studies

#### Types of studies

Randomized controlled trials (RCTs), cohort studies (CSs), and case–control studies (CCSs).

#### Types of participants

Participants were patients with a clear diagnosis of COVID-19, aged ≥ 18 years, regardless of gender and clinical types. We grouped patients according to their clinical types. The classification criteria of clinical types of COVID-19 patients referred to *Diagnosis and Treatment Protocol for COVID-19 Patients (Tentative 8th Edition)* [8]. The clinical symptoms of mild type patients were mild, and there was no evidence of pneumonia on imaging. Moderate type patients presented fever and respiratory symptoms, and chest radiology suggested pneumonia. Adult COVID-19 patients meeting any of the following were regarded as severe type. (1) Tachypnea, respiratory rate ≥ 30 breaths/min; (2) At rest, oxygen saturation ≤ 93% during air suction; (3) Partial pressure of oxygen (PaO2)/fraction of inspired oxygen (FiO2) ≤ 300 mmHg (1 mmHg = 0.133 kPa); PaO2/FiO2 should be corrected by the following formula in high altitude areas (altitude more than 1000 m): PaO2/FiO2 × [760/atmospheric pressure (mmHg)]. (4) Clinical symptoms were gradually worsening, and lung imaging indicated lesion significantly progressed > 50% within 24 ~ 48 h. COVID-19 patients meeting one of the following conditions were classified into critical type. (1) Respiratory failure demanding mechanical ventilation; (2) Shock; (3) Combined with other organ failure and demanded intensive monitoring and treatment.

In this review, included patients were subsumed into four groups according to the clinical types of COVID-19: mild or moderate types into mild group, severe or critical types into severe group, convalescent type into convalescent group, and unknown type or containing two or more of the above three groups into mixed group.

#### Types of interventions

Patients in the intervention/TCM group were treated with TCM or a combination of TCM and conventional Western medicine (CWM). We did not restrict the dosage form of used TCM. Patients in the control/CWM group were treated with CWM (e.g., antiviral treatment, nutritional support, anti-infection treatment).

#### Types of outcome measures

Primary outcomes

(1) Proportion of patients progressing to severe cases.

(2) Mortality rate of severe or critical patients.

Because the outcomes of patients were closely related to clinical types, we used the proportion of patients progressing to severe cases as the primary outcome of mild or moderate patients, and the mortality rate as the primary outcome of severe or critical patients.

Secondary outcomes

(1) Total effective rate. We defined the total effective rate as the proportion of the total number of patients whose clinical symptoms improve ≥ 30%.

(2) Clinical cure rate. We defined the clinical cure of COVID-19 patients as achieving all of the following conditions: no fever > 3 days; significant reduction of respiratory symptoms; chest CT images improved markedly; two consecutive negative 2019-nCoV nucleic acids tests (not on the same day).

(3) Lung CT improvement rate. A reduction of 30% or more in the area of the lesion on the lung CT image was considered to be an improvement in lung CT.

(4) TCM symptom scores. TCM symptom scores were assessed in accordance with *The Guidelines for Clinical Research of New Drugs of Traditional Chinese Medicine*. None, mild, moderate, and severe of the main symptoms corresponded to 0, 2, 4, and 6 points, while none, mild, moderate, and severe of the secondary symptoms corresponded to 0, 1, 2, and 3 points. Calculated the total points.

(5) Disappearance rate and disappearance time of main symptoms (fever, cough, fatigue);

(6) Discharge rate and length of hospital stay.

(7) The rate and conversion time of negative 2019-nCoV nucleic acids tests for two consecutive times (not on the same day).

(8) Incidence of adverse events.

(9) Related inflammatory or immune indicators including white blood cell count (WBC), lymphocyte count (LYM), lymphocyte percentage (LYM%), C-reactive protein (CRP), and interleukin 6 (IL-6).

### Literature search

We systematically searched 7 databases including PubMed, EMBASE, Cochrane Library, CNKI (China National Knowledge Infrastructure), CBM (Chinese Biomedical Database), VIP (VIP Information Database), and WanFang Database from their inception up to July 21, 2021, to identify RCTs, CSs, and CCSs of TCM for COVID-19. We referred to the retrieval method of “P + I + S” and searched with “subject words + free words”. In addition, we added the specific names of commonly used TCM to the search formula to minimize the problem of missed detection. The search terms included “Medicine, Chinese Traditional”, “Traditional Chinese medicine”, “Huoxiang Zhengqi”, “Lianhua Qingwen”, “Qingfei Paidu Decoction”, “Toujie Quwen granules”, “Hanshiyi Formula”, “Coronavirus Disease-19”, “COVID-19”, “2019-nCoV Infection”, “SARS-CoV-2 Infection” and so on. The complete search strategy of seven databases was shown in (Additional file 1). In order to obtain as much literature as possible, we also manually searched related articles and clinical studies.

### Study screening and data extraction

The eligibility of retrieved studies was evaluated using the established inclusion and exclusion criteria. The contents of data extraction include first author, publication year, country or region of the patients, sample size, methodological quality, treatment regimen of intervention and control, basic characteristics (age, gender, clinical type) of the included patients, duration of treatment, outcome measures, and adverse events. Studies without detailed information on outcome measures were excluded. Study screening and data extraction were conducted independently by two reviewers, and any differences were settled by discussion or the third reviewer’s decision.

### Quality assessment

The quality of RCT was evaluated in accordance with the quality assessment criteria from the Cochrane Handbook [9]. The evaluation included the generation of random sequences, allocation concealment, blinding, completeness of outcome data, selective reporting, and other biases. The quality of CS and CCS were evaluated using the corresponding Newcastle–Ottawa Scale (NOS) [10]. The study was scored based on 8 items in three categories: selection of participants, comparability between study groups, and measurement of exposure factors or results. The total score is 9 points. In our study, articles with a score ≥ 7 were designated as high-quality articles. The quality assessment of each included study was independently conducted by two reviewers, and any discrepancies were settled by discussion or the third reviewer’s decision.

### Data analysis

RevMan5.4 was used to perform statistical analysis on the extracted data of the included studies. The relative risk (RR) and weighted mean difference (WMD) were used to analyze dichotomous data and continuous data with 95% confidence interval (CI). When the heterogeneity test I^2^ ≤ 50%, the fixed-effects model was selected for combined analysis, otherwise the random-effects model was used. Evaluation of primary outcome measures and secondary outcome measures was performed separately for each group. Results from RCTs were prioritized when they did not agree with the conclusions drawn from CSs or CCSs.

## Results

### Description of studies

**Fig. 1:**
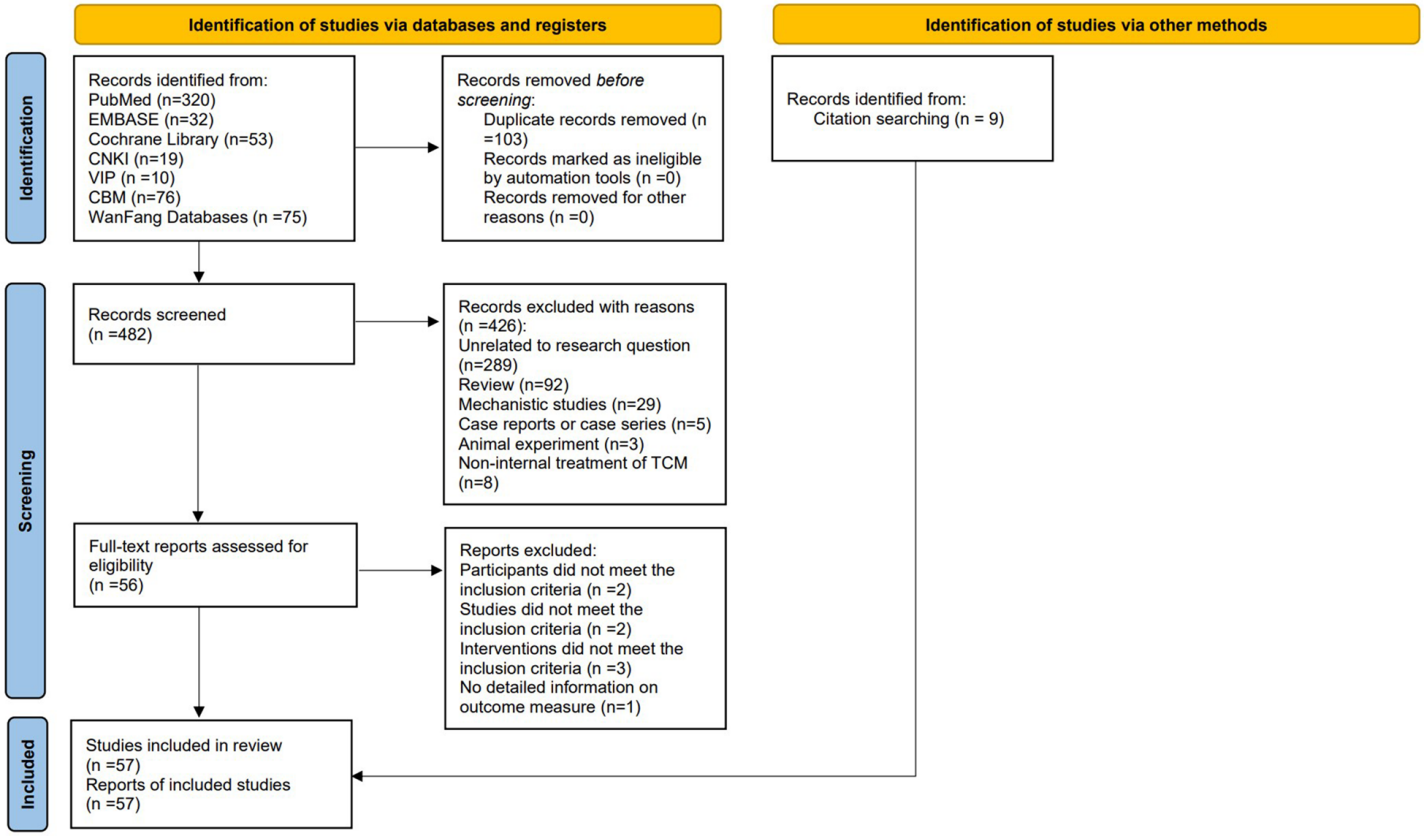
The flow diagram of study screening

The flow diagram of study screening with reference to PRISMA 2020 statement was summarized in Fig. 1.

We retrieved 585 relevant articles from 7 databases, of which 103 were duplicates, and removed 426 articles because they were reviews, case reports, case series, mechanistic studies, or not associated with TCM for COVID-19 after reading titles and abstracts. We also excluded trials in which the intervention was non-internal treatment of TCM, or the patient did not have a definite diagnosis of COVID-19. After intensive full-text reading of the remaining 56 articles, 8 articles were eliminated. Among them, the participants of two studies did not meet the convalescent diagnosis or were younger than 18 years old; The study types of two studies were case series or cross-sectional study; The interventions of three studies were different administration time, rather than TCM treatment or integrated TCM and CWM treatment compared with CWM treatment; One study did not have detailed information on outcome measure. In addition, we searched the citations of relevant articles and included 9 eligible studies. Eventually, we included 57 eligible studies, including 29 RCTs [11–39] and 28 retrospective studies (RSs) [40–67].

The details of 29 RCTs and 28 RSs were provided in (Additional files 2 and 3). Of the 29 RCTs, 15 were subsumed into mild group, 1 into severe group, 11 into mixed group, and 2 into convalescent group. Of the 28 RSs, 12 were subsumed into mild group, 7 into severe group, 8 into mixed group, and 1 into convalescent group. The sample size of 29 RCTs ranged from 20 to 295, with 3060 patients altogether. The sample size of 28 RSs ranged from 22 to 8939, with 12,460 patients altogether.

Lianhua Qingwen capsule (or granule) was the most frequently used TCM in the included studies. It was used in 4 RCTs and 4 RSs, with 394 and 230 cases respectively. Qingfei Paidu decoction was the most widely used TCM. It was used by 1 RCT and 3 RSs, with 70 and 2669 users respectively. Other frequently used TCM included Shufeng Jiedu capsule, “Fei Yan No. 1” formula, Huashi Baidu formula, Reduning injection, Shenhuang granule, etc.

### Assessment of methodological quality

**Fig. 2:**
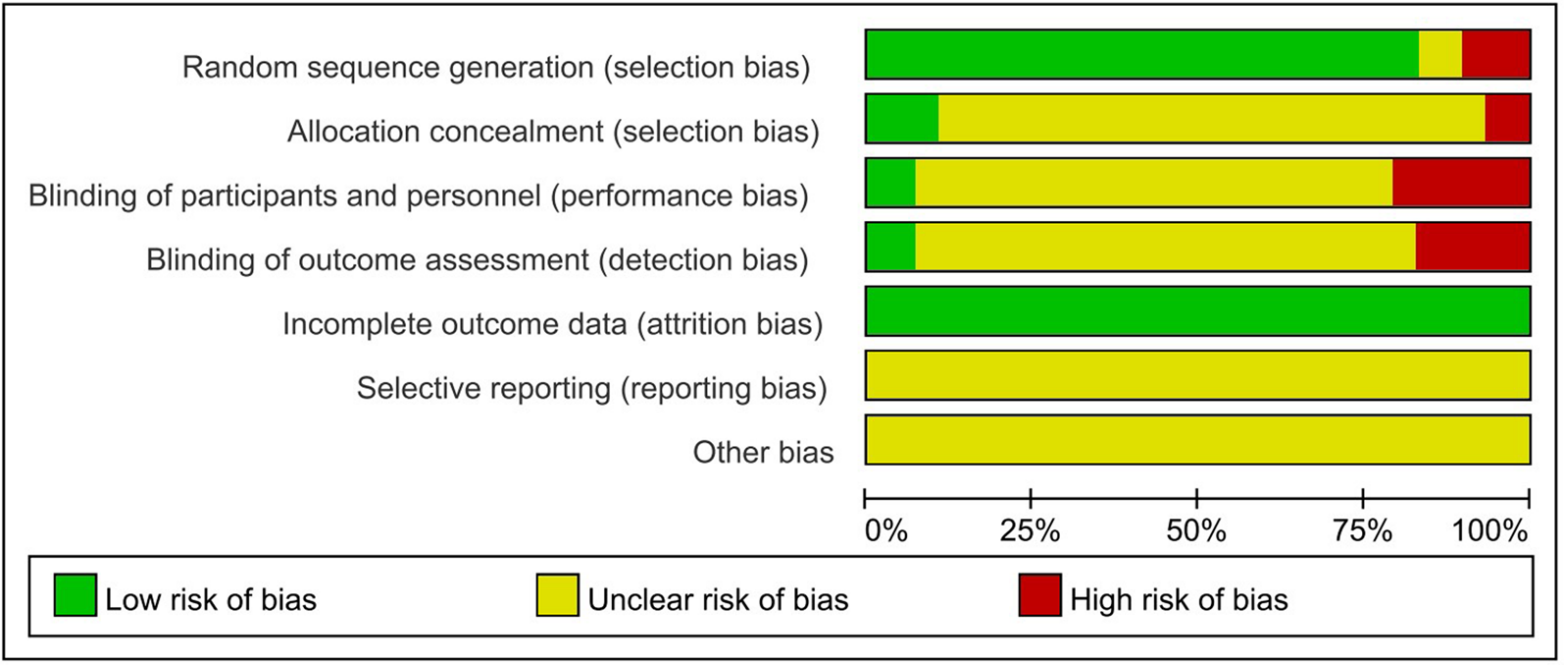
The methodological quality of 29 RCTs

Twenty-four RCTs reported the generation of random sequences, of which three RCTs [12, 26, 29] described the performance of allocation concealment. Two RCTs [12, 29] blinded participants and researchers as well as outcome evaluators. Twenty-nine RCTs completely reported the data of each primary outcome, including lost to follow-up and withdrawal. The information described in 29 RCTs could not enable the reviewers to determine whether the study had reporting bias or other potential bias. The methodological quality of 29 RCTs was summarized in Fig. 2.

Since the 28 RSs included were all CSs, the NOS corresponding to CS was used to evaluate their quality. All RSs had quality scores of no less than 7, of which 16 studies rated 9, 11 studies rated 8, and 1 study rated 7. Overall, the quality of 28 RSs was high. Details of the methodological quality evaluation of the 28 RSs were provided in (Additional file 4).

### Efficacy assessment

#### Proportion of patients progressing to severe cases

**Fig. 3:**
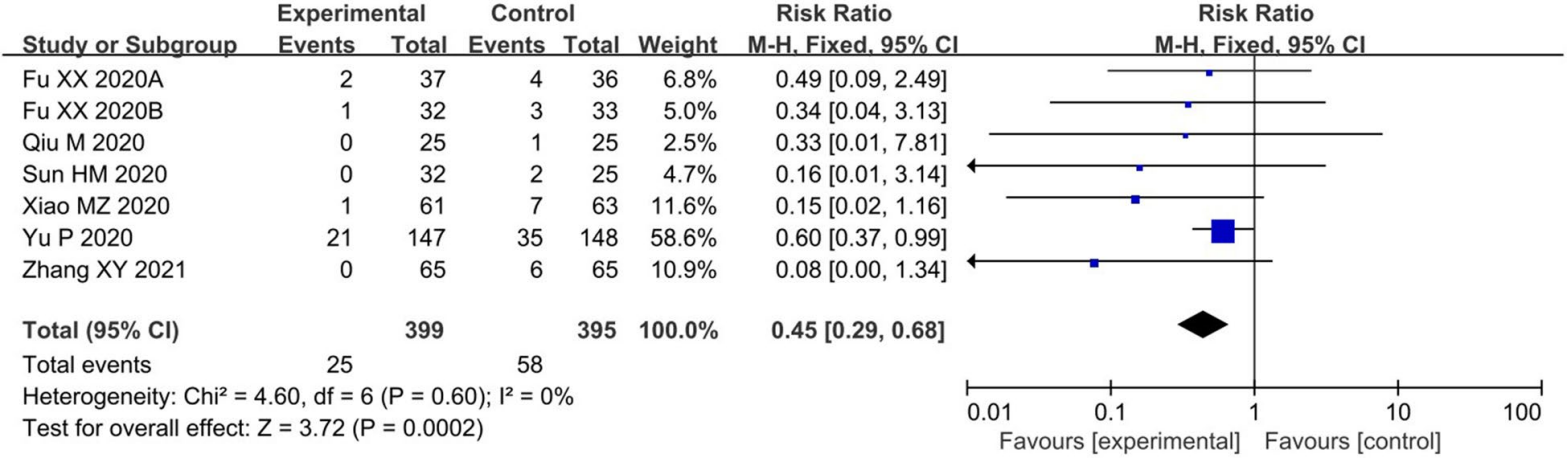
Primary outcome (1) in RCTs

**Fig. 4:**
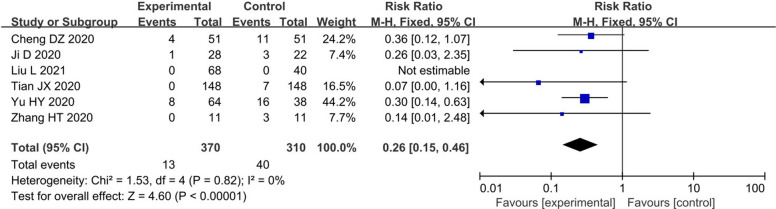
Primary outcome (1) in RSs

The meta-analysis of 7 RCTs [11, 12, 16, 18, 19, 22, 23] demonstrated that TCM could observably lessen the proportion of patients progressing to severe cases [RR = 0.45, 95% CI (0.29, 0.68), I^2^ = 0%, P = 0.0002] (Fig. 3). In addition, 6 RSs [40, 42, 44, 47–49] evaluated this proportion and reached the same conclusion [RR = 0.26, 95% CI (0.15, 0.46), I^2^ = 0%, P < 0.00001] (Fig. 4). Both RCTs and RSs confirmed that TCM could decrease the proportion of patients progressing to severe cases by more than 55%.

#### Mortality rate of severe or critical patients

**Fig. 5:**
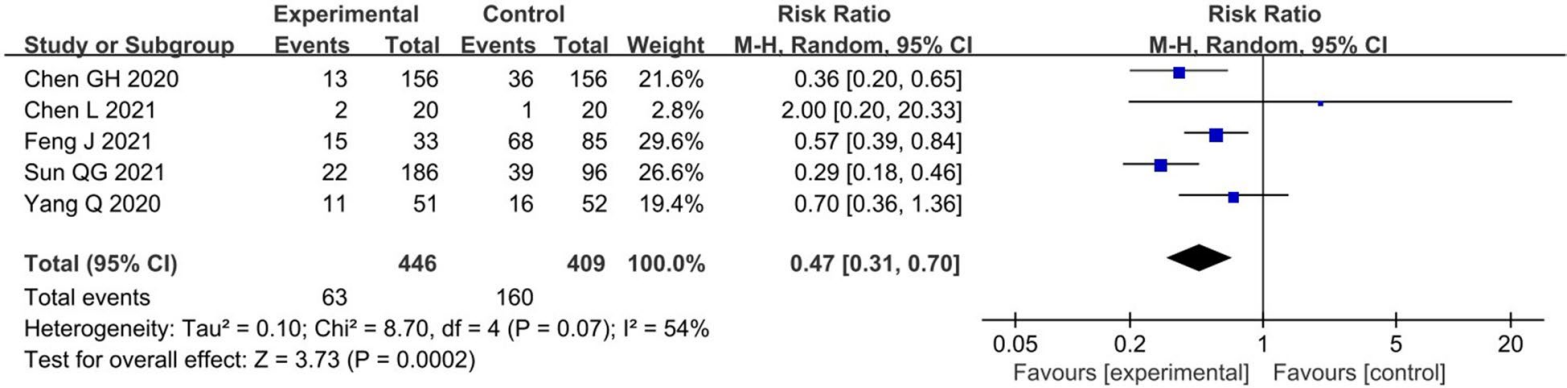
Primary outcome (2) in RSs

A high-quality RCT [26] we included indicated that the mortality rate of severe or critical patients in TCM group was visibly lower compared with CWM group [38.6% (22/57) vs 75.9% (41/54), RR = 0.51, 95% CI (0.35, 0.73), P = 0.0002]. Five RSs [52, 54, 55, 57, 58] reached the same conclusion [RR = 0.47, 95% CI (0.31, 0.70), I^2^ = 54%, P = 0.0002] (Fig. 5). In summary, TCM could decrease the mortality rate of severe or critical patients by more than 49%.

#### Secondary outcomes

Efficacy assessment of secondary outcomes was summarized in (Additional file 5).

Meta-analysis indicated that TCM could enhance the total effective rate by 18%, clinical cure rate by 26%, and lung CT improvement rate by 19%. Applying TCM could reduce TCM symptom scores by more than 2.75 points. The discrepancy in the disappearance rate of fever was not statistically significant, but the disappearance time of fever could be shortened by more than 1.05 days in the TCM group compared with the CWM group. TCM could enhance the disappearance rate of cough by more than 33% and disappearance rate of fatigue by more than 28%, but the difference in the disappearance time of cough and fatigue was not statistically significant. TCM could enhance the discharge rate by 33% and shorten the length of hospital stay by 3.07 days, especially in severe or critical cases. In addition, TCM could increase the rate of negative 2019-nCoV nucleic acids tests by 37%, and shorten the conversion time of negative 2019-nCoV nucleic acids tests by more than 1.58 days. The results of RSs showed that the incidence of adverse events in TCM group was 82% of that in CWM group, but the difference did not reach statistical significance in RCTs. However, it was not observed that TCM increased the incidence of serious adverse events in COVID-19 patients, on the contrary, it has been shown that applying TCM could reduce the incidence of serious adverse events in severe or critical cases [98.1% (53/54) vs 78.9% (45/57), P = 0.002] [26]. Applying TCM could increase WBC by 0.25 × 10^9^/L and LYM by 0.23 × 10^9^/L. In addition, TCM could decrease CRP by 7.65 mg/L and IL-6 by 4.81 ng/L. The difference in LYM% between TCM and CWM group did not reach statistical significance.

### Publication bias

**Fig. 6:**
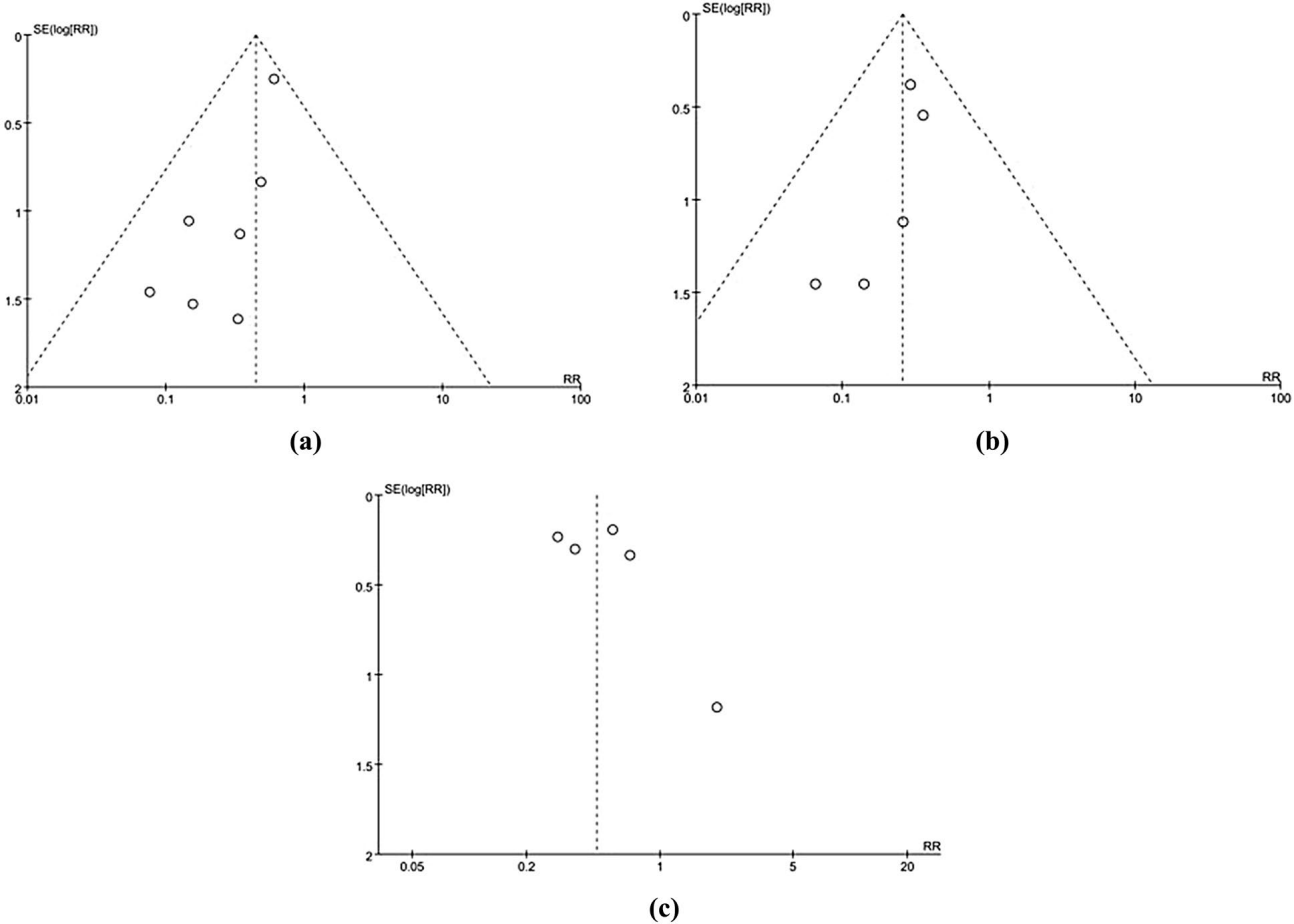
Funnel plots of primary outcomes

We made funnel plots for the two primary outcomes. The asymmetry of the three funnel plots suggested that the study might have a moderate publication bias (Fig. 6). We considered that this might be related to the small number and sample size of the included studies.

## Discussion

### Common formulas for COVID-19

Of the prescriptions used in 57 studies, seven formulas were more commonly used. Lianhua Qingwen Capsule was developed by Academician Yiling Wu in 2003 in order to combat severe acute respiratory syndrome by referring to the ancient Chinese prescriptions Maxing shigan Decoction and Yinqiao Powder. In recent years, it has been widely used in the treatment of various viral influenza [68]. Qingfei Paidu Decoction was innovated by Youwen Ge, a distinguished researcher of China Academy of Chinese Medical Sciences, according to the core pathogenesis of COVID-19 and the prescriptions in *Treatise on Cold Damage Diseases*, including Maxing Shigan Decoction, Shegan Mahuang Decoction, Xiaochaihu Decoction, Wuling Powder, etc. [69]. Shufeng Jiedu Capsule was a Chinese patent medicine made by Hunan Medical University by improving the ancestral secret prescription "Qudu powder" of Tujia Nationality in Western Hunan Province, China. Shufeng Jiedu Capsule was approved by China food and Drug Administration in 2009, and then widely used as antiviral, antibacterial, antitumor and anti-inflammatory drugs [70]. Reduning injection, made from Lonicerae Japonicae Flos (金银花), Artemisiae Annuae Herba (青蒿), and Gardeniae Fructus (栀子), was approved by China Food and Drug Administration in 2005 and has been shown to have antibacterial, antiviral, antipyretic and antipyretic effects [71]. "Pneumonia No.1 Formula", Huashi Baidu Formula, Shenhuang Granule and other drugs were new prescriptions developed by TCM experts for the treatment of COVID-19 based on their many years of clinical experience and combined with the clinical characteristics of COVID-19 [69].

### Common herbs for COVID-19

According to the frequency statistics of the Chinese medicinal herbs used in 57 included studies, we obtained 99 herbs that appeared twice or more. The details were shown in (Additional file 6). The 20 most frequently used herbs were Glycyrrhizae Radix et Rhizoma (甘草), Armeniacae Semen Amarum (苦杏仁), Ephedrae Herba (麻黄), Gypsum fibrosum (石膏), Poria (茯苓), Forsythiae Fructus (连翘), Scutellariae Radix (黄芩), Pinelliae Rhizoma Praeparatum (法半夏), Pogostemonis Herba (广藿香), Lonicerae Japonicae Flos (金银花), Bupleuri Radix (柴胡), Citri Reticulatae Pericarpium (陈皮), Atractylodis Macrocephalae Rhizoma (白术), Rhei Radix et Rhizoma (大黄), Magnoliae Officinalis Cortex (厚朴), Isatidis Radix (板蓝根), Tsaoko Fructus (草果), Menthae Haplocalycis Herba (薄荷), Zingiberis Rhizoma Recens (生姜) and Dryopteridis Crassirhizomatis Rhizoma (绵马贯众).

### Core components and targets for COVID-19

In addition, we reviewed the literature and sorted out the components and targets of TCM for COVID-19 predicted by molecular docking. According to the mechanism of action, the targets were divided into antiviral targets and anti-inflammatory or immune-regulating targets. Antiviral targets included ACE2, 3CLpro, Spro, Plpro, Rdrp, and Nsp14. Anti-inflammatory or immune-regulating targets included IL-6, TNF, IL1B, CCL2, and AKT1.

We predicted that 149 components had therapeutic effects on COVID-19 by molecular docking, of which 20 components had good docking scores not only with antiviral targets but also with anti-inflammatory or immune-regulating targets. The 20 components were formononetin, naringenin, bicuculline, luteolin, quercetin, astragaloside IV, kaempferol, isoquercitrin, rutin, beta-carotene, salvigenin, indirubin, baicalein, calycosin, artemetin, wogonin, rosmarinic acid, apigenin, 7-methoxy-2-methyl isoflavone and emodin [72–94]. The details of components and targets were shown in Additional files 7 and 8].

#### Antiviral targets

Studies suggested that 2019-nCoV infection was initiated through the combination of virus with host cell surface receptor ACE2 (angiotensin converting enzyme 2), fusion of virus with cell membrane, and release of virus genome into cells. Among them, Spro (viral spike protein) mediated the activity of receptor binding and membrane fusion [95, 96]. 3CLpro (3C-like protease) in coronaviruses played a vital role in advancing the polyprotein translated from viral RNA [97]. RdRp (RNA-dependent RNA polymerase) was essential for coronavirus replication and transcription and might be a primary target for antiviral drugs [98]. Viral Plpro (papain-like cysteine protease) was considered an important target of antiviral drugs because it was required for SARS-CoV-2 replication and could promote the dysregulation of signaling cascades in infected cells [99]. Nsp14 (nonstructural protein 14) was a functional enzyme related to replication fidelity and involved in mRNA capping, which played an essential role in virus replication [100].

#### Anti-inflammatory or immune-regulating targets

It has been suggested that a proportion of severe COVID-19 patients might suffer from cytokine storm syndrome and might die because of high inflammation driven by the virus [101]. IL-6 (interleukin-6) was an important cytokine with a variety of physiological functions, including regulating immune cell proliferation and differentiation [102]. IL1B (interleukin 1 beta) was considered an essential pro-inflammatory cytokine related to the origination and development of acute respiratory distress syndrome (ARDS) [103]. TNF (tumor necrosis factor) was regarded as a primary inflammatory cytokine that could drive cytokine production, cell survival, or cell death [104]. CCL2 [Chemokine (C–C motif) ligand 2] exhibited a chemotactic activity for monocytes and was one of the key chemokines regulating monocyte/macrophage migration and infiltration [105]. Severe COVID-19 manifested as ARDS with elevated pro-inflammatory cytokines, involving TNF-α, IL-6, IL1B, and CCL2. Therefore, the treatment related to anti-cytokine or anti-cytokine-signaling would be conducive to the prognosis of COVID-19 [106]. Akt1 was one of 3 related serine/threonine-protein kinases implicated in pulmonary fibrosis and lung injury and also played an essential role in immune cell modulation [72].

#### Core components

It has been confirmed that four flavonoids including formononetin, apigenin, luteolin, and kaempferol had in vitro activities against enterovirus 71 infection due to reducing viral replication and protein synthesis [107]. Studies suggested that naringenin might be a promising treatment strategy against COVID-19 due to its antiviral and anti-inflammatory effects [108]. Bicuculline showed in vitro anti-inflammatory activity and attenuated inflammation by decreasing the generation of pro-inflammatory cytokines, like IL1B and TNF-α, and promoting the production of the anti-inflammatory cytokine interleukin-10 [109, 110]. Quercetin manifested antiviral, anti-inflammatory, and immune-enhancing effects in vitro and some animal models [111]. Astragaloside IV could improve immunologic function of RAW264.7 cells through stimulating the NF-κB/MAPK signaling pathway [112]. Isoquercitrin could inhibit herpes simplex virus-1 replication to exhibit an antiviral effect [113]. Rutin exerted the antiviral effect mainly by inhibiting or modifying various viral proteins such as viral neuraminidase and DNA/RNA polymerase [114]. Beta-Carotene exerted anti-inflammatory activity through suppressing the production and expression of inflammatory mediators in lipopolysaccharide-stimulated RAW264.7 cells and macrophages [115]. Rosmarinic acid and salvigenin had anti-inflammatory activity, and the mechanisms included inhibiting the maturation and release of IL-1β [116]. Indirubin exhibited potent anti-inflammatory activity and could significantly downregulate the generation of IL-6, IL1B, and TNF-α [117]. It has been shown that baicalein exerted anti-H5N1 effects through inhibiting the replication of the influenza H5N1 virus and interfering with the H5N1-induced production of IL-6 and TNF-α in macrophages [118]. Studies suggested that calycosin could diminish the levels of TNF-α, IL-6, and IL1B in mice with acute pancreatitis [119]. Artemetin exhibited evident anti-inflammatory activity in many experimental models in rats [120]. Wogonin exerted antiviral effects against herpes simplex virus (HSV) infection by inhibiting viral replication, mRNA transcription, and protein synthesis [121]. 7-Methoxy-2-methyl isoflavone might exert therapeutic effect on COVID-19 through inhibiting inflammatory storms and modulating immune function [90]. Emodin had anti-inflammatory, antiviral, and antibacterial effects, and the anti-inflammatory had been confirmed in various inflammatory models, including asthma, arthritis, and pancreatitis [122].

.

### Advantages and disadvantages of TCM for COVID-19

TCM has been used to treat epidemic diseases in China for thousands of years. Our study including 29 RCTs and 28 RSs systematically evaluated the efficacy of TCM in treating COVID-19. The results demonstrated that applying TCM treatment could evidently decrease the proportion of patients progressing to severe cases and the mortality rate of severe or critical patients. Moreover, our analysis indicated that the results of total effective rate, clinical cure rate, lung CT improvement rate, TCM symptom scores, disappearance time of fever, disappearance rate of cough and fatigue, discharge rate, length of hospital stay, the rate and conversion time of negative 2019-nCoV nucleic acids tests, incidence of adverse events, LYM, CRP and IL-6 in TCM group were better compared to CWM group.

TCM for COVID-19 had the characteristics of multiple components and multiple targets, which played a role from multiple aspects and had important therapeutic significance throughout the disease. However, TCM also had certain disadvantages. For example, TCM formulas had many components and targets, and were not targeted when exerting therapeutic effect, so it was difficult to elaborate its mechanism in depth.

### Differences from previous meta-analyses of TCM for COVID-19

In addition, it is worth noting that the prevention and treatment goals for different clinical types of patients with COVID-19 are different. For mild or moderate types patients, reducing the proportion progressing to severe cases is the main goal. For severe or critical patients, reducing mortality rate is the main goal. For convalescent type patients, reducing the positive RT-PCR test results and alleviating clinical symptoms is the main goal [123]. At present, the published systematic reviews of TCM for COVID-19 have not classified patients according to the clinical types, and this review systematically appraised the treatment of TCM in different clinical types of COVID-19 for the first time.

In our study, the primary outcomes were generally recognized and reasonable. The study participants were grouped according to clinical types. Moreover, we included not only RCTs but also RSs, which confirmed the efficacy of TCM for COVID-19 from different angles and levels. Furthermore, our study conducted a systematic literature review on the mechanism of TCM for COVID-19 based on 57 included studies.

### Limitations and perspective

However, this review also has some limitations. Firstly, the quality of some included studies is not high or the sample size is small, so bias is prone to occur. Secondly, due to the need for grouping evaluation based on clinical types, the number of included studies is still relatively small. Thirdly, there are too few clinical studies in the recovery period to see the long-term effects of TCM for COVID-19. Fourthly, in terms of mechanism, most researches focus on network pharmacology and cell level, and there is too little evidence at the animal level and organizational level. Therefore, more high-quality and large-sample RCTs are demanded to assess the efficacy of TCM for COVID-19, and more animal experiments are required to verify the efficacy.

The epidemic of COVID-19 is still rampant worldwide, and the pace of virus mutation has not stopped. It is still necessary to find effective drugs against COVID-19, and we believe that TCM is a module worthy of further research in the treatment of COVID-19. Our study made a more systematic review on the efficacy of TCM for COVID-19 and discussed the possible mechanisms, which provided clinical reference and theoretical basis for further research on the mechanism of TCM for COVID-19.

## Conclusion

TCM had a definite therapeutic effect on COVID-19. Especially, TCM could decrease the proportion of patients progressing to severe cases by 55% and the mortality rate of severe or critical patients by 49%. Furthermore, the mechanism was explored, and it was concluded that TCM played a therapeutic role in COVID-19 mainly through anti-virus, anti-inflammation, and regulation of immunity.

## Supplementary Information

**Additional file 1.** Search strategy of seven databases.

**Additional file 2.** Basic characteristics of included RCTs.

**Additional file 3.** Basic characteristics of included retrospective studies.

**Additional file 4.** The quality of the included retrospective studies.

**Additional file 5.** Efficacy assessment of secondary outcomes.

**Additional file 6.** The frequency of the Chinese medicinal herbs.

**Additional file 7.** Antiviral components and targets.

**Additional file 8.** Anti-inflammatory or immune-regulating components and targets.

## Data Availability

The datasets used and/or analysed during the current study are available from the corresponding author on reasonable request.

## Abbreviations

2019n-CoV: 2019 Novel coronavirus
3CLpro: 3C-like protease
ACE2: Angiotensin converting enzyme 2
ARDS: Acute respiratory distress syndrome
CCL2: Chemokine (C–C motif) ligand 2
CCSs: Case–control studies
CI: Confidence interval
COVID-19: Coronavirus disease 2019
CRP: C-reactive protein
CSs: Cohort studies
CWM: Conventional Western medicine
FiO2: Fraction of inspired oxygen
IL1B: Interleukin 1 beta
IL-6: Interleukin 6
LYM%: Lymphocyte percentage
LYM: Lymphocyte count
Nsp14: Nonstructural protein 14
PaO2: Partial pressure of oxygen
Plpro: Papain-like protease
RCTs: Randomized controlled trials
RdRp: RNA-dependent RNA polymerase
RR: Relative risk
RSs: Retrospective studies
SARS-CoV-2: Severe acute respiratory syndrome coronavirus 2
Spro: Spike protein
TCM: Traditional Chinese medicine
TNF: Tumor necrosis factor
WBC: White blood cell count
WMD: Weighted mean difference
