# Supplementary material for: Efficacy and mechanisms of traditional Chinese medicine for COVID-19: a systematic review
Source: Chin Med. 2022 Feb 28;17:30. doi: 10.1186/s13020-022-00587-7 (PMC8883015; doi:10.1186/s13020-022-00587-7)
Supplement: Supplementary file 1 — Additional file 1. Search strategy of seven databases. [file 13020_2022_587_MOESM1_ESM.docx]

**Additional file 1. Search strategy of seven databases**

**English search strategy**

**PubMed**

1. COVID 19[Title/Abstract]
2. COVID-19 Virus Disease[Title/Abstract]
3. COVID 19 Virus Disease[Title/Abstract]
4. COVID-19 Virus Diseases[Title/Abstract]
5. Disease, COVID-19 Virus[Title/Abstract]
6. Virus Disease, COVID-19[Title/Abstract]
7. COVID-19 Virus Infection[Title/Abstract]
8. COVID 19 Virus Infection[Title/Abstract]
9. COVID-19 Virus Infections[Title/Abstract]
10. Infection, COVID-19 Virus[Title/Abstract]
11. Virus Infection, COVID-19[Title/Abstract]
12. 2019-nCoV Infection[Title/Abstract]
13. 2019 nCoV Infection[Title/Abstract]
14. 2019-nCoV Infections[Title/Abstract]
15. Infection, 2019-nCoV[Title/Abstract]
16. Coronavirus Disease-19[Title/Abstract]
17. Coronavirus Disease 19[Title/Abstract]
18. 2019 Novel Coronavirus Disease[Title/Abstract]
19. 2019 Novel Coronavirus Infection[Title/Abstract]
20. 2019-nCoV Disease[Title/Abstract]
21. 2019 nCoV Disease[Title/Abstract]
22. 2019-nCoV Diseases[Title/Abstract]
23. Disease, 2019-nCoV[Title/Abstract]
24. COVID19[Title/Abstract]
25. Coronavirus Disease 2019[Title/Abstract]
26. Disease 2019, Coronavirus[Title/Abstract]
27. SARS Coronavirus 2 Infection[Title/Abstract]
28. SARS-CoV-2 Infection[Title/Abstract]
29. Infection, SARS-CoV-2[Title/Abstract]
30. SARS CoV 2 Infection[Title/Abstract]
31. SARS-CoV-2 Infections[Title/Abstract]
32. COVID-19 Pandemic[Title/Abstract]
33. COVID 19 Pandemic[Title/Abstract]
34. COVID-19 Pandemics[Title/Abstract]
35. Pandemic, COVID-19[Title/Abstract]
36. 1 or 2 or 3 or 4 or 5 or 6 or 7 or 8 or 9 or 10 or 11 or 12 or 13 or 14 or 15 or 16 or 17 or 18 or 19 or 20 or 21 or 22 or 23 or 24 or 25 or 26 or 27 or 28 or 29 or 30 or 31 or 32 or 33 or 34 or 35 or 36
37. Medicine, Chinese Traditional[Title/Abstract]
38. Traditional Chinese Medicine[Title/Abstract]
39. Chung I Hsueh[Title/Abstract]
40. Hsueh, Chung I[Title/Abstract]
41. Traditional Medicine, Chinese[Title/Abstract]
42. Zhong Yi Xue[Title/Abstract]
43. Chinese Traditional Medicine[Title/Abstract]
44. Chinese Medicine, Traditional[Title/Abstract]
45. Traditional Tongue Diagnosis[Title/Abstract]
46. Tongue Diagnoses, Traditional[Title/Abstract]
47. Tongue Diagnosis, Traditional[Title/Abstract]
48. Traditional Tongue Diagnoses[Title/Abstract]
49. Traditional Tongue Assessment[Title/Abstract]
50. Tongue Assessment, Traditional[Title/Abstract]
51. Traditional Tongue Assessments[Title/Abstract]
52. Huoxiang Zhengqi[Title/Abstract]
53. Lianhua Qingwen[Title/Abstract]
54. Shuanghuanglian oral liquid[Title/Abstract]
55. Shufeng Jiedu capsules[Title/Abstract]
56. Jinhua Qinggan granules[Title/Abstract]
57. Qingfei Paidu Decoction[Title/Abstract]
58. Toujie Quwen granules[Title/Abstract]
59. Pneumonia No.1 Formula[Title/Abstract]
60. Huashi Baidu[Title/Abstract]
61. Xuanfei Baidu[Title/Abstract]
62. Hanshiyi Formula[Title/Abstract]
63. Maxing Shigan Decoction[Title/Abstract]
64. Yinqiao Powder[Title/Abstract]
65. Reduning[Title/Abstract]
66. Tanreqing[Title/Abstract]
67. Reyanning[Title/Abstract]
68. Xiyanping[Title/Abstract]
69. Xuebijing[Title/Abstract]
70. Buzhong Yiqi[Title/Abstract]
71. Shengmai Powder[Title/Abstract]
72. 37 or 38 or 39 or 40 or 41 or 42 or 43 or 44 or 45 or 46 or 47 or 48 or 49 or 50 or 51 or 52 or 53 or 54 or 55 or 56 or 57 or 58 or 59 or 60 or 61 or 62 or 63 or 64 or 65 or 66 or 67 or 68 or 69 or 70 or 71
73. randomized controlled trial[Publication Type]
74. randomized[Title/Abstract]
75. placebo[Title/Abstract]
76. 73 or 74 or 75
77. Cohort Studies[Title/Abstract]
78. Cohort Study[Title/Abstract]
79. Studies, Cohort[Title/Abstract]
80. Study, Cohort[Title/Abstract]
81. Concurrent Studies[Title/Abstract]
82. Studies, Concurrent[Title/Abstract]
83. Concurrent Study[Title/Abstract]
84. Study, Concurrent[Title/Abstract]
85. Closed Cohort Studies[Title/Abstract]
86. Cohort Studies, Closed[Title/Abstract]
87. Closed Cohort Study[Title/Abstract]
88. Cohort Study, Closed[Title/Abstract]
89. Study, Closed Cohort[Title/Abstract]
90. Studies, Closed Cohort[Title/Abstract]
91. Analysis, Cohort[Title/Abstract]
92. Cohort Analysis[Title/Abstract]
93. Analyses, Cohort[Title/Abstract]
94. Cohort Analyses[Title/Abstract]
95. Historical Cohort Studies[Title/Abstract]
96. Cohort Study, Historical[Title/Abstract]
97. Historical Cohort Study[Title/Abstract]
98. Study, Historical Cohort[Title/Abstract]
99. Cohort Studies, Historical[Title/Abstract]
100. Studies, Historical Cohort[Title/Abstract]
101. Incidence Studies[Title/Abstract]
102. Incidence Study[Title/Abstract]
103. Studies, Incidence[Title/Abstract]
104. Study, Incidence[Title/Abstract]
105. 77 or 78 or 79 or 80 or 81 or 82 or 83 or 84 or 85 or 86 or 87 or 88 or 89 or 90 or 91 or 92 or 93 or 94 or 95 or 96 or 97 or 98 or 99 or 100 or 101 or 102 or 103 or 104
106. Case-Control Studies[Title/Abstract]
107. Case-Control Study[Title/Abstract]
108. Studies, Case-Control[Title/Abstract]
109. Study, Case-Control[Title/Abstract]
110. Case-Comparison Studies[Title/Abstract]
111. Case Comparison Studies[Title/Abstract]
112. Case-Comparison Study[Title/Abstract]
113. Studies, Case-Comparison[Title/Abstract]
114. Study, Case-Comparison[Title/Abstract]
115. Case-Compeer Studies[Title/Abstract]
116. Studies, Case-Compeer[Title/Abstract]
117. Case-Referrent Studies[Title/Abstract]
118. Case Referrent Studies[Title/Abstract]
119. Case-Referrent Study[Title/Abstract]
120. Studies, Case-Referrent[Title/Abstract]
121. Study, Case-Referrent[Title/Abstract]
122. Case-Referent Studies[Title/Abstract]
123. Case Referent Studies[Title/Abstract]
124. Case-Referent Study[Title/Abstract]
125. Studies, Case-Referent[Title/Abstract]
126. Study, Case-Referent[Title/Abstract]
127. Case-Base Studies[Title/Abstract]
128. Case Base Studies[Title/Abstract]
129. Studies, Case-Base[Title/Abstract]
130. Case Control Studies[Title/Abstract]
131. Case Control Study[Title/Abstract]
132. Studies, Case Control[Title/Abstract]
133. Study, Case Control[Title/Abstract]
134. Nested Case-Control Studies[Title/Abstract]
135. Case-Control Studies, Nested[Title/Abstract]
136. Case-Control Study, Nested[Title/Abstract]
137. Nested Case Control Studies[Title/Abstract]
138. Nested Case-Control Study[Title/Abstract]
139. Studies, Nested Case-Control[Title/Abstract]
140. Study, Nested Case-Control[Title/Abstract]
141. Matched Case-Control Studies[Title/Abstract]
142. Case-Control Studies, Matched[Title/Abstract]
143. Case-Control Study, Matched[Title/Abstract]
144. Matched Case Control Studies[Title/Abstract]
145. Matched Case-Control Study[Title/Abstract]
146. Studies, Matched Case-Control[Title/Abstract]
147. Study, Matched Case-Control[Title/Abstract]
148. 106 or 107 or 108 or 109 or 110 or 111 or 112 or 113 or 114 or 115 or 116 or 117 or 118 or 119 or 120 or 121 or 122 or 123 or 124 or 125 or 126 or 127 or 128 or 129 or 130 or 131 or 132 or 133 or 134 or 135 or 136 or 137 or 138 or 139 or 140 or 141 or 142 or 143 or 144 or 145 or 146 or 147
149. 76 or 105 or 148
150. 36 and 72 and 149

**Cochrane Library**

#1 MeSH descriptor: [COVID-19] explode all trees

#2 (COVID 19):ti,ab,kw OR (COVID-19 Virus Disease):ti,ab,kw OR (COVID 19 Virus Disease):ti,ab,kw OR (COVID-19 Virus Diseases):ti,ab,kw OR (Disease, COVID-19 Virus):ti,ab,kw OR (Virus Disease, COVID-19):ti,ab,kw OR (COVID-19 Virus Infection):ti,ab,kw OR (COVID 19 Virus Infection):ti,ab,kw OR (COVID-19 Virus Infections):ti,ab,kw OR (Infection, COVID-19 Virus):ti,ab,kw OR (Virus Infection, COVID-19):ti,ab,kw OR (Coronavirus Disease-19):ti,ab,kw OR (Coronavirus Disease 19):ti,ab,kw OR (2019 Novel Coronavirus Disease):ti,ab,kw OR (2019 Novel Coronavirus Infection):ti,ab,kw OR (COVID19):ti,ab,kw OR (Coronavirus Disease 2019):ti,ab,kw OR (Disease 2019, Coronavirus):ti,ab,kw OR (SARS Coronavirus 2 Infection):ti,ab,kw OR (SARS-CoV-2 Infection):ti,ab,kw OR (Infection, SARS-CoV-2):ti,ab,kw OR (SARS CoV 2 Infection):ti,ab,kw OR (SARS-CoV-2 Infections):ti,ab,kw OR (COVID-19 Pandemic):ti,ab,kw OR (COVID 19 Pandemic):ti,ab,kw OR (COVID-19 Pandemics):ti,ab,kw OR (Pandemic, COVID-19):ti,ab,kw

#3 #1 or #2

#4 MeSH descriptor: [Medicine, Chinese Traditional] explode all trees

#5 (Traditional Chinese Medicine):ti,ab,kw OR (Chung I Hsueh):ti,ab,kw OR (Hsueh, Chung I):ti,ab,kw OR (Traditional Medicine, Chinese):ti,ab,kw OR (Zhong Yi Xue):ti,ab,kw OR (Chinese Traditional Medicine):ti,ab,kw OR (Chinese Medicine, Traditional):ti,ab,kw OR (Traditional Tongue Diagnosis):ti,ab,kw OR (Tongue Diagnoses, Traditional):ti,ab,kw OR (Tongue Diagnosis, Traditional):ti,ab,kw OR (Traditional Tongue Diagnoses):ti,ab,kw OR (Traditional Tongue Assessment):ti,ab,kw OR (Tongue Assessment, Traditional):ti,ab,kw OR (Traditional Tongue Assessments):ti,ab,kw OR (Huoxiang Zhengqi):ti,ab,kw OR (Lianhua Qingwen):ti,ab,kw OR (Shuanghuanglian oral liquid):ti,ab,kw OR (Shufeng Jiedu capsules):ti,ab,kw OR (Jinhua Qinggan granules):ti,ab,kw OR (Qingfei Paidu Decoction):ti,ab,kw OR (Toujie Quwen granules):ti,ab,kw OR (Pneumonia No.1 Formula):ti,ab,kw OR (Huashi Baidu):ti,ab,kw OR (Xuanfei Baidu):ti,ab,kw OR (Hanshiyi Formula):ti,ab,kw OR (Maxing Shigan Decoction):ti,ab,kw OR (Yinqiao Powder):ti,ab,kw OR (Reduning):ti,ab,kw OR (Tanreqing):ti,ab,kw OR (Reyanning):ti,ab,kw OR (Xiyanping):ti,ab,kw OR (Xuebijing):ti,ab,kw OR (Buzhong Yiqi):ti,ab,kw OR (Shengmai Powder):ti,ab,kw

#6 #4 or #5

#7 #3 and #6

**EMBASE**

#1 'coronavirus disease 2019'/exp

#2 'covid-19':ab,ti OR 'covid 19':ab,ti OR 'covid-19 virus disease':ab,ti OR 'covid 19 virus disease':ab,ti OR 'covid-19 virus diseases':ab,ti OR 'disease, covid-19 virus':ab,ti OR 'virus disease, covid-19':ab,ti OR 'covid-19 virus infection':ab,ti OR 'covid 19 virus infection':ab,ti OR 'covid-19 virus infections':ab,ti OR 'infection, covid-19 virus':ab,ti OR 'virus infection, covid-19':ab,ti OR '2019-ncov infection':ab,ti OR '2019 ncov infection':ab,ti OR '2019-ncov infections':ab,ti OR 'infection, 2019-ncov':ab,ti OR 'coronavirus disease-19':ab,ti OR 'coronavirus disease 19':ab,ti OR '2019 novel coronavirus disease':ab,ti OR '2019 novel coronavirus infection':ab,ti OR '2019-ncov disease':ab,ti OR '2019 ncov disease':ab,ti OR '2019-ncov diseases':ab,ti OR 'disease, 2019-ncov':ab,ti OR 'covid19':ab,ti OR 'coronavirus disease 2019':ab,ti OR 'disease 2019, coronavirus':ab,ti OR 'sars coronavirus 2 infection':ab,ti OR 'sars-cov-2 infection':ab,ti OR 'infection, sars-cov-2':ab,ti OR 'sars cov 2 infection':ab,ti OR 'sars-cov-2 infections':ab,ti OR 'covid-19 pandemic':ab,ti OR 'covid 19 pandemic':ab,ti OR 'covid-19 pandemics':ab,ti OR 'pandemic, covid-19':ab,ti

3# 1# OR 2#

4# 'chinese medicine'/exp

5# 'medicine, chinese traditional':ab,ti OR 'traditional chinese medicine':ab,ti OR 'chung i hsueh':ab,ti OR 'hsueh, chung i':ab,ti OR 'traditional medicine, chinese':ab,ti OR 'zhong yi xue':ab,ti OR 'chinese traditional medicine':ab,ti OR 'chinese medicine, traditional':ab,ti OR 'traditional tongue diagnosis':ab,ti OR 'tongue diagnoses, traditional':ab,ti OR 'tongue diagnosis, traditional':ab,ti OR 'traditional tongue diagnoses':ab,ti OR 'traditional tongue assessment':ab,ti OR 'tongue assessment, traditional':ab,ti OR 'traditional tongue assessments':ab,ti OR ' Huoxiang Zhengqi ':ab,ti OR ' Lianhua Qingwen ':ab,ti OR ' Shuanghuanglian oral liquid ':ab,ti OR ' Shufeng Jiedu capsules ':ab,ti OR ' Jinhua Qinggan granules ':ab,ti OR ' Qingfei Paidu Decoction ':ab,ti OR ' Toujie Quwen granules ':ab,ti OR ' Pneumonia No.1 Formula ':ab,ti OR ' Huashi Baidu ':ab,ti OR ' Xuanfei Baidu ':ab,ti OR ' Hanshiyi Formula ':ab,ti OR ' Maxing Shigan Decoction ':ab,ti OR ' Yinqiao Powder ':ab,ti OR ' Reduning ':ab,ti OR ' Tanreqing ':ab,ti OR ' Reyanning ':ab,ti OR ' Xiyanping ':ab,ti OR ' Xuebijing ':ab,ti OR ' Buzhong Yiqi ':ab,ti OR ' Shengmai Powder ':ab,ti

6# 4# OR 5#

7# 'random':ab,ti OR 'placebo':ab,ti OR 'double-blind':ab,ti

8# 'cohort analysis'/exp

9# 'cohort studies':ab,ti OR 'cohort study':ab,ti OR 'studies, cohort':ab,ti OR 'study, cohort':ab,ti OR 'concurrent studies':ab,ti OR 'studies, concurrent':ab,ti OR 'concurrent study':ab,ti OR 'study, concurrent':ab,ti OR 'closed cohort studies':ab,ti OR 'cohort studies, closed':ab,ti OR 'closed cohort study':ab,ti OR 'cohort study, closed':ab,ti OR 'study, closed cohort':ab,ti OR 'studies, closed cohort':ab,ti OR 'analysis, cohort':ab,ti OR 'cohort analysis':ab,ti OR 'analyses, cohort':ab,ti OR 'cohort analyses':ab,ti OR 'historical cohort studies':ab,ti OR 'cohort study, historical':ab,ti OR 'historical cohort study':ab,ti OR 'study, historical cohort':ab,ti OR 'cohort studies, historical':ab,ti OR 'studies, historical cohort':ab,ti OR 'incidence studies':ab,ti OR 'incidence study':ab,ti OR 'studies, incidence':ab,ti OR 'study, incidence':ab,ti

10# 8# OR 9#

11# 'case control study'/exp

12# 'case-control studies':ab,ti OR 'case-control study':ab,ti OR 'studies, case-control':ab,ti OR 'study, case-control':ab,ti OR 'case-comparison studies':ab,ti OR 'case comparison studies':ab,ti OR 'case-comparison study':ab,ti OR 'studies, case-comparison':ab,ti OR 'study, case-comparison':ab,ti OR 'case-compeer studies':ab,ti OR 'studies, case-compeer':ab,ti OR 'case-referrent studies':ab,ti OR 'case referrent studies':ab,ti OR 'case-referrent study':ab,ti OR 'studies, case-referrent':ab,ti OR 'study, case-referrent':ab,ti OR 'case-referent studies':ab,ti OR 'case referent studies':ab,ti OR 'case-referent study':ab,ti OR 'studies, case-referent':ab,ti OR 'study, case-referent':ab,ti OR 'case-base studies':ab,ti OR 'case base studies':ab,ti OR 'studies, case-base':ab,ti OR 'case control studies':ab,ti OR 'case control study':ab,ti OR 'studies, case control':ab,ti OR 'nested case-control studies':ab,ti OR 'case-control studies, nested':ab,ti OR 'case-control study, nested':ab,ti OR 'nested case control studies':ab,ti OR 'nested case-control study':ab,ti OR 'studies, nested case-control':ab,ti OR 'study, case control':ab,ti OR 'study, nested case-control':ab,ti OR 'matched case-control studies':ab,ti OR 'case-control studies, matched':ab,ti OR 'case-control study, matched':ab,ti OR 'matched case control studies':ab,ti OR 'matched case-control study':ab,ti OR 'studies, matched case-control':ab,ti OR 'study, matched case-control':ab,ti

13# 11# OR 12#

14# 7# OR 10# OR 13#

15# 3# AND 6# AND 14#

**Chinese search strategy**

**CNKI**

(主题=新冠肺炎 + 新型冠状病毒肺炎 + 新冠病毒 + 新型冠状病毒) AND (主题=中医药 + 中药 + 中草药 + 草药 + 中医 + 藿香正气 + 连花清瘟 + 双黄连口服液 + 疏风解毒胶囊 + 金花清感颗粒 + 清肺排毒汤 + 透解祛瘟颗粒 + 肺炎1号方 + 化湿败毒 + 宣肺败毒 + 寒湿疫方 + 麻杏石甘汤 + 银翘散 + 热毒宁 + 痰热清 + 热炎宁 + 喜炎平 + 血必净+ 补中益气+ 生脉散) AND (主题=随机 + 随机对照 + 病例对照 + 队列研究)

**WangFang Database**

主题:(新冠肺炎 or 新型冠状病毒肺炎or 新冠病毒 or 新型冠状病毒) and 主题:(中医药 or 中药 or中草药 or 草药 or 中医or 藿香正气 or 连花清瘟 or 双黄连口服液 or 疏风解毒胶囊 or 金花清感颗粒 or 清肺排毒汤 or 透解祛瘟颗粒 or 肺炎1号方 or 化湿败毒 or 宣肺败毒 or 寒湿疫方 or 麻杏石甘汤 or 银翘散 or 热毒宁 or 痰热清 or 热炎宁 or 喜炎平 or 血必净or 补中益气or 生脉散) and 主题:(随机 or 随机对照 or 队列研究 or 病例对照)

**VIP Information Database**

摘要=新冠肺炎+新型冠状病毒肺炎+新冠病毒+新型冠状病毒AND 摘要=中医药+中药+中草药+草药+中医+藿香正气+连花清瘟+双黄连口服液+疏风解毒胶囊+金花清感颗粒+清肺排毒汤+透解祛瘟颗粒+肺炎1号方+化湿败毒+宣肺败毒+寒湿疫方+麻杏石甘汤+银翘散+热毒宁+痰热清+热炎宁+喜炎平+血必净+补中益气+生脉散 AND 摘要=随机+随机对照+病例对照+队列研究

**CBM**

1. "新冠肺炎"[常用字段:智能] OR "新型冠状病毒肺炎"[常用字段:智能] OR "新冠病毒"[常用字段:智能] OR "新型冠状病毒"[常用字段:智能]

2. "中医药"[常用字段:智能] OR "中药"[常用字段:智能] OR "中草药"[常用字段:智能] OR "草药"[常用字段:智能] OR "中医"[常用字段:智能] OR "藿香正气"[常用字段:智能] OR "连花清瘟"[常用字段:智能] OR "双黄连口服液"[常用字段:智能] OR "疏风解毒胶囊"[常用字段:智能] OR "金花清感颗粒"[常用字段:智能] OR "清肺排毒汤"[常用字段:智能] OR "透解祛瘟颗粒"[常用字段:智能] OR "肺炎1号方"[常用字段:智能] OR "化湿败毒"[常用字段:智能] OR "宣肺败毒"[常用字段:智能] OR "寒湿疫方"[常用字段:智能] OR "麻杏石甘汤"[常用字段:智能] OR "银翘散"[常用字段:智能] OR "热毒宁"[常用字段:智能] OR "痰热清"[常用字段:智能] OR "热炎宁"[常用字段:智能] OR "喜炎平"[常用字段:智能] OR "血必净"[常用字段:智能] OR "补中益气"[常用字段:智能] OR "生脉散"[常用字段:智能]

3. "随机"[常用字段:智能] OR "随机对照"[常用字段:智能] OR "病例对照"[常用字段:智能] OR "队列研究"[常用字段:智能]

4. (#3) AND (#2) AND (#1)
