# Supplementary material for: Efficacy and mechanisms of traditional Chinese medicine for COVID-19: a systematic review
Source: Chin Med. 2022 Feb 28;17:30. doi: 10.1186/s13020-022-00587-7 (PMC8883015; doi:10.1186/s13020-022-00587-7)
Supplement: Supplementary file 2 — Additional file 2. Basic characteristics of included RCTs. [file 13020_2022_587_MOESM2_ESM.docx]

Additional file 2. Basic characteristics of included RCTs

| Study | country or region | Sample size (randomized/analyzed) | Age | Gender (M/F) | Clinical type | Intervention | Control | Duration | Outcome measures |
| --- | --- | --- | --- | --- | --- | --- | --- | --- | --- |
| Xiao MZ 2020 [11] | Wuhan, Hubei Province, China | T:61/61 C:63/63 | T:56.07±12.10 C:53.90±13.92 | T:33/28 C:35/28 | mild | C+ Huoxiang Zhengqi dropping pills (one bag, twice a day) and Lianhua Qingwen granules (one bag, three times a day) | CWM | 14d | (1)(7)(9)(11) |
| Zhang XY 2021 [12] | Jiangxi Province, China | T:65/65 C:65/65 | T:44.31±13.45 C:48.25±14.22 | T:32/33 C:28/37 | mild and moderate | C+ XYP (10 mg/kg once per day, maximum daily dosage 500 mg） | CWM | 7-14d | (1)(8)(10) |
| Shi NN 2021 [13] | Wuhan, Hubei  province, China | T:20/20 C:20/20 | T:50.3±17.7 C:54.8±19.3 | T:7/13 C:18/2 | mild and moderate | C+Huashi Baidu Formula, 137g bid | Lopinavir-Ritonavir, 500 mg bid | From admission to discharg | (3)(14)(17)(18)(19)(21)(22) |
| Duan C 2020 [14] | Wuhan, Hubei Province, China | T:82/82 C:41/41 | T:51.99±13.88 C:50.29±13.17 | T:39/43 C:23/18 | mild | C+Jinhua Qinggan granules ( 10g tid ) | CWM | 5d | (6)(7)(9)(11)(17) |
| He Q 2020 [15] | Wuhan, Hubei Province, China | T:36/36 C:36/35 | 15-82 | unclear | mild | C + Buzhong Yiqi Decoction, 1 dose per day | Abidol 200 mg tid | 10d | (4)(6)(16)(17)(21)(22) |
| Fu XX 2020A [16] | Guangzhou, Guangdong Province, China | T:37/35 C:36/32 | T:45.26±7.25 C:44.68±7.45 | T:19/18 C:19/17 | moderate | C+Toujie Quwen granules (one bag, twice a day) | Arbidol tablets (0.2g, tid) and ambroxol tablets (30mg, tid) | 15d | (1)(3)(14)(17)(18)(19)(20)(21) |
| Liu W 2021 [17] | Wuhan, Hubei Province, China | T:44/44 C:44/44 | T:48.51±4.56 C:48.43±4.52 | T:16/28 C:15/29 | mild | C+Lianhua Qingwen Capsules 0.4g tid and pneumonia No.2 formula 1 dose per day | CWM+Abidol 0.2g tid + Oseltamivir 15 mg bid | 21d | (3)(4)(17) |
| Qiu M 2020 [18] | Chongqing, China | T:25/25 C:25/25 | T:53.35±18.35 C:51.32±14.62 | T:13/12 C:14/11 | moderate | C+Maxing Xuanfei Jiedu decoction, 150ml, tid | α-Interferon 5 million U, atomization inhalation, bid +Wei Lituo lopinavir ritonavir tablets (each tablet contains 200 mg of lopinavir and 50 mg of ritonavir), 2 tablets bid | 10d | (1)(5)(6)(8)(10) |
| Sun HM 2020 [19] | Hebei Province, China | T:32/32 C:25/25 | T:45.4±14.10 C:42.0±11.70 | T:17/15 C:11/14 | mild and moderate | C + Lianhua Qingke granules, 1 bag, tid | CWM | 14d | (1)(5)(7)(9)(11) |
| Wang L 2020 [20] | Jingzhou, Hubei Province, China | T:40/40 C:40/40 | T:41.1±14.5 C:40.8±13.7 | T:23/27 C:28/12 | moderate | Shengmai Powder combined with Shenlingbaizhu Powder, 1 dose per day | CWM | From admission to discharg | (3)(5)(6)(8)(10)(12)(15)(17)(18)(19)(21) |
| Wang Y 2021 [21] | Xiangyang, Hubei Province, China | T:70/70 C:70/70 | T:48±13.2 C:49.4±13.3 | T:35/35 C:36/34 | moderate | C+Qingfei Paidu Decoction, 1 dose per day | CWM | 10d | (3)(5)(6)(13)(17)(18)(19)(20) |
| Yu P 2020 [22] | Wuhan, Hubei Province, China | T:147/128 C:148/108 | T:48.27±9.56 C:47.25±8.67 | T:82/65 C:89/59 | mild and moderate | C+Lianhua Qingwen granules, 6g tid | Abidol Hydrochloride Dispersible Tablets 0.2g tid; Moxifloxacin Hydrochloride Tablets 0.4g qd; Ambroxol Hydrochloride Tablets 30 mg tid; | 7d | (1)(3)(5)(17)(18)(20)(21) |
| Fu XX 2020B [23] | Guangzhou, Guangdong Province, China | T:32/31 C:33/30 | T:43.26±7.15 C:43.68±6.45 | T:17/15 C:19/14 | mild and moderate | Toujie Quwen granules, 1 dose per day + Moxifloxacin Tablets, 0.4g, qd + Ambroxol Tablets, 30mg, tid | Abidol Tablets, 0.2g, tid; Moxifloxacin Tablets, 0.4g, qd; Ambroxol Tablets, 30 mg, tid; | 10d | (1)(3)(5)(17)(18)(19)(20)(21) |
| Xiao Q 2020 [24] | Wuhan, Hubei Province, China | T:100/100 C:100/100 | T:60.90±8.70 C:62.20±7.50 | T:64/36 C:66/34 | mild and moderate | C + Shufeng Jiedu capsules, 4 capsules, tid | Abidol Tablets, 0.2g tid | 14d | (3)(5)(8)(10)(12)(17)(18)(20) |
| Yang MB 2020 [25] | Xi'an/Hubei Province, China | T:26/26 C:23/23 | T:50.35±13.37 C:47.17±16.57 | T:16/10 C:9/14 | moderate | C + Reyanning Mixture, oral, 10-20ml/time, 2-4 times a day. | CWM | 7d | (5)(6)(16)(17)(21) |
| Zhou S 2021 [26] | Hubei Province, China | T:61/57 C:61/54 | 64.7±12.0 | T:33/24 C:38/16 | severe and critical | C+Shenhuang Granule | CWM | 14d | (2)(3)(17) |
| Ma QH 2021 [27] | Jiangsu/Hubei Province, China | T:27/27 C:23/23 | T:49.7±16.0 C:51.5±15.9 | T:16/11 C:12/11 | mild and severe | C+ReDuNing injection (RDN) | CWM | 14d | (3)(17) |
| Ni L 2021 [28] | Anhui/Heilongjiang/Jiangsu/Hubei Province | T:59/59 C:59/59 | T:52.5±16.3 C:51.5±20.1 | T:27/32 C:25/34 | mild, moderate and severe | C+Shuanghuanglian oral liquids（60 mL, three times daily） | CWM | 14d | (3)(16)(17) |
| Wang JB 2020 [29] | Beijing, China | T:24/24 C:23/23 | T:46.8±14.4 C:51.4±17.6 | T:14/10 C:12/11 | unclear | C+Keguan-1 drug (twice daily) | CWM | 14d | (8)(15) |
| Xu XL 2021 [30] | Hubei/Jiangsu/Guangdong/Henan/Beijing/Tianjin Province, China | T:77/77 C:80/80 | T:49.1±15.7 C:50.4±16.0 | T:43/34 C:44/36 | mild, moderate and severe | C+ Reduning injection, 20 mL/day | CWM | 14d | (4)(17) |
| Hu K 2021 [31] | Hubei/Shanghai/Guangdong/Hebei/Beijing/Tianjin/Zhejiang Province, China | T:142/142 C:142/142 | T:50.4±15.2 C:51.8±14.8 | T:79/63 C:71/71 | unclear | C+LH capsules (4 capsules thrice daily) | CWM | 14d | (4)(5)(8)(10)(12)(15)(16)(17) |
| Ai XY 2020 [32] | Guangzhou, Guangdong Province, China | T:55/55 C:43/43 | T:43.98±12.6 C:45.95±18.3 | T:24/31 C:17/26 | mild, moderate and severe | C + "Pneumonia No.1 Formula" granules, 1 bag tid | CWM | 12d | (3)(4)(6)(13)(17)(19) |
| Wang LQ 2020 [33] | Wuhan, Hubei Province, China | T:58/58 C:60/60 | T:63.1±12.7 C:57.6±19.9 | T:29/29 C:34/26 | mild, moderate and severe | C + Gegen Qinlian Pills, 1 bag, tid | CWM | From admission to discharg | (6)(7)(8)(9)(11)(14)(15)(17)(18)(19)(21) |
| Zheng ZZ 2020 [34] | Wuhan, Hubei Province, China | T:65/65 C:65/65 | T:17-84 C:18-85 | T:42/23 C:44/21 | moderate and severe | C + Chinese herbal decoction, 1 dose per day | CWM | 14d | (3)(4) |
| Ding XJ 2020 [35] | Wuhan, Hubei Province, China | T:51/51 C:49/49 | T:54.7±21.3 C:50.8±23.5 | T:39/12 C:39/10 | mild, moderate, severe and critical | C + Qingfei Tongxie Fuzheng decoction, 1 dose per day | CWM | 10d | (5)(7)(9)(17)(21)(22) |
| Liao GR [36] | Yunnan Province, China | T:35/35 C:35/35 | T:65.25±7.42 C:67.16±8.64 | T:20/15 C:18/17 | unclear | C + Chinese herbal decoction, 1 dose per day | CWM | 7d | (7)(9)(11)(17) |
| Zheng WJ [37] | Jingzhou, Hubei Province, China | T:40/40 C:40/40 | T:50.38±5.25 C:50.21±5.38 | T:24/16 C:25/15 | unclear | C + Sulfotanshinone Sodium Injection, intravenous drip, once daily | CWM | 10d | (3)(21)(22) |
| Shi SF 2020 [38] | Hubei/Jiangsu Province, China | T:30/30 C:30/30 | T:50.93±10.83 C:50.23±10.67 | T:11/19 C:11/19 | convalescent | Yiqi Yangyin granules, 2 bags, bid | Normal diet | 14d | (3)(4)(6)(17) |
| Zhao F 2020 [39] | Xi'an Province, China | T:38/38 C:14/14 | T:47.53±13.15 C:47.57±14.81 | T:20/18 C:9/5 | convalescent | Xuanfei Dayu decoction, 1 dose per day | Normal diet | 15d | (5)(18)(19) |

(1) Proportion of patients progressing to severe cases; (2) Mortality rate of severe or critical patients; (3) Total effective rate; (4) Clinical cure rate; (5) Lung CT improvement rate; (6) TCM symptom scores; (7) Disappearance rate of fever; (8) Disappearance time of fever; (9) Disappearance rate of cough; (10) Disappearance time of cough; (11) Disappearance rate of fatigue; (12) Disappearance time of fatigue; (13) Discharge rate; (14) Length of hospital stay; (15) The rate of negative 2019-nCoV nucleic acids tests; (16) The conversion time of negative 2019-nCoV nucleic acids tests; (17) Incidence of adverse events; (18) white blood cell count (WBC count); (19) Lymphocyte count (LYM count); (20) Lymphocyte percentage (LYM%); (21) C-reactive protein (CRP); (22) Interleukin 6 (IL-6).

**References**

1. Xiao M, Tian J, Zhou Y, Xu X, Min X, Lv Y, et al. Efficacy of Huoxiang Zhengqi dropping pills and Lianhua Qingwen granules in treatment of COVID-19: A randomized controlled trial. *Pharmacol Res.* 2020;**161**:105126.
2. Zhang XY, Lv L, Zhou YL, Xie LD, Xu Q, Zou XF, et al. Efficacy and safety of Xiyanping injection in the treatment of COVID-19: A multicenter, prospective, open-label and randomized controlled trial. *Phytother Res.* 2021;**35**:4401-10.
3. Shi N, Guo L, Liu B, Bian Y, Chen R, Chen S, et al. Efficacy and safety of Chinese herbal medicine versus Lopinavir-Ritonavir in adult patients with coronavirus disease 2019: A non-randomized controlled trial. *Phytomedicine.* 2021;**81**:153367.
4. Duan C, Xia WG, Zheng CJ, Sun GB, Li ZL, Li QL. et al. Clinical observation on Jinhua Qinggan Granule combined with conventional western medicine therapy in treating mild cases of coronavirus disease 2019. *Journal of Traditional Chinese Medicine.* 2020; **61**:1473-1477.
5. He Q, Zhang QJ, Gan XW, Li XG. Clinical analysis of Buzhong Yiqi Decoction in treating mild cases of coronavirus disease 2019. *Journal of Emergency in Traditional Chinese Medicine.* 2021; **30**: 385-387.
6. Fu XX, Lin LP, Tan XH. Clinical study on 37 cases of COVID-19 treated with integrated traditional Chinese and western medicine. *Traditional Chinese Drug Research and Clinical Pharmacology.* 2020; **31**: 600-604.
7. Liu W, Su XY, Liao XL. Effect of antiviral drugs combined with traditional Chinese medicine on mild cases of coronavirus disease 2019. *Contemporary Medical Symposium.* 2021; **19**:159-160.
8. Qiu M, Li QT, Zhu DP, Wang CH, Sun QZ, Qian CF. et al. Efficacy observation of Maxing Xuanfei Jiedu Decoction on moderate COVID-19. *Journal of Emergency in Traditional Chinese Medicine.* 2020; **29**: 1129-1130 + 1132.
9. Sun HM, Xu F, Zhang L ,Wei C, Chen JY, Wang QX. et al. Study on clinical efficacy of Lianhua Qingke Granule in treatment of mild and ordinary COVID-19. *Chinese Journal of Experimental Traditional Medical Formulae.* 2020; **26**: 29-34.
10. Wang L, Xu M, Wang Y, Li HB, Liu N, Zuo, JL. Clinical study on Shengmai Powder combined with Shenlingbaizhu Powder in the treatment of common coronavirus disease 2019. *China Journal of Traditional Chinese Medicine and Pharmacy.* 2020; **35**: 4268-4271.
11. Wang Y, Chen Li, Zheng L, Ku BQ, Yu R, Zhang XF. Clinical effects of Qingfei Paidu Decoction combined with conventional treatment on Patients with coronavirus disease 2019. *Chinese Traditional Patent Medicine.* 2021; **43**: 656-659.
12. Yu P, Li YZ, Wang SB, Wang Y. Effect of Lianhua Qingwen Granules plus Abidol on treatment of mild coronavirus disease 2019. *Chinese Pharmaceutical Journal.* 2020; **55**: 1042-1045.
13. Fu XX, Lin LP, Tan XH. Clinical observation on effect of Toujie Quwen Granules in treatment of COVID-19. *Chinese Journal of Experimental Traditional Medical Formulae.* 2020; **26**: 44-48.
14. Xiao Q, Jiang YJ, Wu SS, Wang Y, An J, Xu WP. et al. Analysis of the value of Shufeng Jiedu Capsule combined with Abidol in the treatment of mild coronavirus disease 2019. *Journal of Emergency in Traditional Chinese Medicine.* 2020; **29**: 756-758.
15. Yang MB, Dang SS, Huang S, Li YJ, Guo YL. Multi-center clinical observation of Reyanning Mixture in treatment of COVID-19. *Chinese Journal of Experimental Traditional Medical Formulae.* 2020; **26**: 7-12.
16. Zhou S, Feng J, Xie Q, Huang T, Xu X, Zhou D, et al. Traditional Chinese medicine shenhuang granule in patients with severe/critical COVID-19: A randomized controlled multicenter trial. *Phytomedicine.* 2021;**89**:153612.
17. Ma Q, Xie Y, Wang Z, Lei B, Chen R, Liu B, et al. Efficacy and safety of ReDuNing injection as a treatment for COVID-19 and its inhibitory effect against SARS-CoV-2. *J Ethnopharmacol.* 2021;**279**:114367.
18. Ni L, Wen Z, Hu X, Tang W, Wang H, Zhou L, et al. Effects of Shuanghuanglian oral liquids on patients with COVID-19: a randomized, open-label, parallel-controlled, multicenter clinical trial. *Front Med.* 2021;**15**:704-17.
19. Wang JB, Wang ZX, Jing J, Zhao P, Dong JH, Zhou YF, et al. Exploring an Integrative Therapy for Treating COVID-19: A Randomized Controlled Trial. *Chin J Integr Med.* 2020;**26**:648-55.
20. Xu X, Zhang J, Zheng W, Yang Z, Zhao X, Wang C, et al. Efficacy and safety of Reduning injection in the treatment of COVID-19: a randomized, multicenter clinical study. *Ann Palliat Med.* 2021;**10**:5146-55.
21. Hu K, Guan WJ, Bi Y, Zhang W, Li L, Zhang B, et al. Efficacy and safety of Lianhuaqingwen capsules, a repurposed Chinese herb, in patients with coronavirus disease 2019: A multicenter, prospective, randomized controlled trial. *Phytomedicine.* 2021;**85**:153242.
22. Ai XY, Luo C, Lin LP, Xie M, Fang HM, Tan XH. Therapeutic effect of integrated traditional Chinese and western medicine on COVID-19 in Guangzhou. *China Tropical Medicine.* 2020; **20**: 746-750.
23. Wang LQ, Li WN, Huang W, Zhou ZM, Deng YL, Hu YL. et al. Clinical study of Gegen Qinlian pill in treating COVID-19. *Modernization of Traditional Chinese Medicine and Materia Medica-World Science and Technology.* 2020; **22**: 3509-3514.
24. Zheng ZZ, Bai ZG, Li CJ, Ge SP, Luo Y, He GD. Observation on the effect of TCM Syndrome Differentiation and Treatment for COVID-19. *Medical Journal of Communications.* 2020; **34**: 117-118.
25. Ding XJ, Zhang Y, He DC, Zhang MY, Tan YJ, Yu AR. et al. Clinical effect and mechanism of Qingfei Touxie Fuzheng Recipe in the treatment of COVID-19. *Herald of Medicine.* 2020; **39**: 640-644.
26. Liao GR. Efficacy and safety of Chinese Herbal Decoction in patients with COVID-19. *International Infections Diseases(Electronic Edition).* 2020; **9**: 353.
27. Zheng WJ, Guan JW. Clinical observation of sodium tanshinone ⅱ A sulfonate in the treatment of COVID-19. *World Latest Medicine Information.* 2020; **20**:267-268.
28. Shi SF, Fang ZY, Xiong K, Ye DL, Wang WM, Wu H. et al. Clinical studies of comprehensive TCM treatment to 30 cases of Qi Yin Deficiency Type of COVID-19 in its recovery period. *Jiangsu Journal of Traditional Chinese Medicine.* 2021; **53**: 25-28.
29. Zhao F, Yang Z, Liu SX, Lv WZ, Lv SX, Mao MH. et al. Clinical study of Xuanfei Dayu Decoction in treating the recovery stage of COVID-19. *Shaanxi Journal of Traditional Chinese Medicine.* 2020; **41**: 846-848.
