# Supplementary material for: Efficacy and mechanisms of traditional Chinese medicine for COVID-19: a systematic review
Source: Chin Med. 2022 Feb 28;17:30. doi: 10.1186/s13020-022-00587-7 (PMC8883015; doi:10.1186/s13020-022-00587-7)
Supplement: Supplementary file 4 — Additional file 4. The quality of the included retrospective studies. [file 13020_2022_587_MOESM4_ESM.docx]

Additional file 4. The quality of the included retrospective studies

| Study | Representativeness of the exposed group | Selection of the nonexposed group | Ascertainment of exposure | Outcome of interest not present at the start of study | Comparability of study groups | Assessment of outcome | Follow-up duration | Adequacy of follow-up of study | Total score |
| --- | --- | --- | --- | --- | --- | --- | --- | --- | --- |
| Tian JX 2020 [40] | 1 | 1 | 1 | 1 | 2 | 1 | 1 | 1 | 9 |
| Xin SY 2020 [41] | 1 | 1 | 1 | 1 | 1 | 1 | 1 | 1 | 8 |
| Zhang HT 2020 [42] | 1 | 1 | 1 | 1 | 2 | 1 | 1 | 1 | 9 |
| Zhang X 2020 [43] | 1 | 1 | 1 | 1 | 1 | 1 | 1 | 1 | 8 |
| Liu L 2021 [44] | 1 | 1 | 1 | 1 | 2 | 1 | 1 | 1 | 9 |
| Shen P 2021 [45] | 1 | 1 | 1 | 1 | 1 | 1 | 1 | 1 | 8 |
| Chen J 2020 [46] | 1 | 1 | 1 | 1 | 2 | 1 | 1 | 1 | 9 |
| Cheng DZ 2020 [47] | 1 | 1 | 1 | 1 | 2 | 1 | 1 | 1 | 9 |
| Ji D 2020 [48] | 1 | 1 | 1 | 1 | 2 | 0 | 1 | 1 | 8 |
| Yu HY 2020 [49] | 1 | 1 | 1 | 1 | 2 | 1 | 1 | 1 | 9 |
| Yao KT 2020 [50] | 1 | 1 | 1 | 1 | 1 | 1 | 1 | 1 | 8 |
| Zhai XK 2020 [51] | 1 | 1 | 1 | 1 | 2 | 1 | 1 | 1 | 9 |
| Sun QG 2021 [52] | 1 | 1 | 1 | 1 | 1 | 1 | 1 | 1 | 8 |
| Wang Y 2021A [53] | 1 | 1 | 1 | 1 | 1 | 1 | 1 | 1 | 8 |
| Chen GH 2020 [54] | 1 | 1 | 1 | 1 | 2 | 1 | 1 | 1 | 9 |
| Feng J 2021 [55] | 1 | 1 | 1 | 1 | 2 | 1 | 1 | 1 | 9 |
| Hu HB 2021 [56] | 1 | 1 | 1 | 1 | 2 | 1 | 1 | 1 | 9 |
| Yang Q 2020 [57] | 1 | 1 | 1 | 1 | 2 | 1 | 1 | 1 | 9 |
| Chen L 2021 [58] | 1 | 1 | 1 | 0 | 1 | 1 | 1 | 1 | 7 |
| Shu ZX 2021 [59] | 1 | 1 | 1 | 1 | 2 | 1 | 1 | 1 | 9 |
| Zhang LH 2021 [60] | 1 | 1 | 1 | 1 | 1 | 1 | 1 | 1 | 8 |
| Ai ZZ 2020 [61] | 1 | 1 | 1 | 1 | 2 | 1 | 1 | 1 | 9 |
| Hu YQ 2020 [62] | 1 | 1 | 1 | 1 | 2 | 1 | 1 | 1 | 9 |
| Lian J 2020 [63] | 1 | 1 | 1 | 1 | 1 | 1 | 1 | 1 | 8 |
| Wang LQ 2021 [64] | 1 | 1 | 1 | 1 | 1 | 1 | 1 | 1 | 8 |
| Shi J 2020 [65] | 1 | 1 | 1 | 1 | 2 | 1 | 1 | 1 | 9 |
| Xia WG 2020 [66] | 1 | 1 | 1 | 1 | 2 | 1 | 1 | 1 | 9 |
| An YW 2021 [67] | 1 | 1 | 1 | 1 | 1 | 1 | 1 | 1 | 8 |

**References**

1. Tian J, Yan S, Wang H, Zhang Y, Zheng Y, Wu H, et al. Hanshiyi Formula, a medicine for Sars-CoV2 infection in China, reduced the proportion of mild and moderate COVID-19 patients turning to severe status: A cohort study. *Pharmacol Res.* 2020;**161**:105127.
2. Xin S, Cheng X, Zhu B, Liao X, Yang F, Song L, et al. Clinical retrospective study on the efficacy of Qingfei Paidu decoction combined with Western medicine for COVID-19 treatment. *Biomed Pharmacother.* 2020;**129**:110500.
3. Zhang HT, Huang MX, Liu X, Zheng XC, Li XH, Chen GQ, et al. Evaluation of the Adjuvant Efficacy of Natural Herbal Medicine on COVID-19: A Retrospective Matched Case-Control Study. *Am J Chin Med.* 2020;**48**:779-92.
4. Zhang X, Xue Y, Chen X, Wu JM, Su ZJ, Sun M, et al. Effects of Tanreqing Capsule on the negative conversion time of nucleic acid in patients with COVID-19: A retrospective cohort study. *J Integr Med.* 2021;**19**:36-41.
5. Liu L, Shi F, Tu P, Chen C, Zhang M, Li X, et al. Arbidol combined with the Chinese medicine Lianhuaqingwen capsule versus arbidol alone in the treatment of COVID-19. *Medicine (Baltimore).* 2021;**100**:e24475.
6. Shen P, Li J, Tu S, Wu Y, Peng Y, Chen G, et al. Positive effects of Lianhuaqingwen granules in COVID-19 patients: A retrospective study of 248 cases. *J Ethnopharmacol.* 2021;**278**:114220.
7. Chen J, Lin S, Niu C, and Xiao Q. Clinical evaluation of Shufeng Jiedu Capsules combined with umifenovir (Arbidol) in the treatment of common-type COVID-19: a retrospective study. *Expert Rev Respir Med.* 2021;**15**:257-65.
8. Cheng DZ, Wang WJ, Li Y, Wu XD, Zhou B, Song QY. Analysis of curative effect of 51 patients with novel coronavirus pneumonia treated with Chinese medicine Lianhua Qingwen: a multicenter retrospective study. *Tianjin Journal of Traditional Chinese Medicine.* 2020; **37**: 509-516.
9. Ji D, Feng P, Fei XY. Retrospective study of clinical efficacy of integrated traditional Chinese and western medicine in treatment of COVID-19. *Shandong Journal of Traditional Chinese Medicine.* 2020; **39**: 645-647 + 653.
10. Yu HY, Ren XH, Qi XX, Zuo Q, Liu D. Efficacy study of Abidol, Qingfei Paidu Decoction, Lianhua Qingwen Capsule and Jinye Baidu Granule in the treatment of mild/moderate COVID-19 in a Fangcang shelter hospital. *Pharmacology and Clinics of Chinese Materia Medica.* 2020; **36**: 2-6.
11. Yao KT, Liu MY, Li X, Huang JH, Cai HB. Retrospective clinical analysis on treatment of coronavirus disease 2019 with traditional Chinese medicine Lianhua Qingwen. *Chinese Journal of Experimental Traditional Medical Formulae.* 2020; **26**: 8-12.
12. Zhai XK, Hao SL, Ma JH, Wei GY, Song KY, Tang C, et al. Observation on clinical effect of Shufeng Jiedu Capsule combined with Abidol Hydrochloride in treatment of COVID-19. *Chinese Traditional and Herbal Drugs.* 2020; **51**: 1167-1170.
13. Sun QG, An XD, Xie P, Jiang B, Tian JX, Yang Q, et al. Traditional Chinese Medicine Decoctions Significantly Reduce the Mortality in Severe and Critically Ill Patients with COVID-19: A Retrospective Cohort Study. *Am J Chin Med.* 2021;**49**:1063-92.
14. Wang Y, Lu C, Li H, Qi W, Ruan L, Bian Y, et al. Efficacy and safety assessment of severe COVID-19 patients with Chinese medicine: A retrospective case series study at early stage of the COVID-19 epidemic in Wuhan, China. *J Ethnopharmacol.* 2021;**277**:113888.
15. Chen G, Su W, Yang J, Luo D, Xia P, Jia W, et al. Chinese herbal medicine reduces mortality in patients with severe and critical Coronavirus disease 2019: a retrospective cohort study. *Front Med.* 2020;**14**:752-9.
16. Feng J, Fang B, Zhou D, Wang J, Zou D, Yu G, et al. Clinical Effect of Traditional Chinese Medicine Shenhuang Granule in Critically Ill Patients with COVID-19: A Single-Centered, Retrospective, Observational Study. *J Microbiol Biotechnol.* 2021;**31**:380-6.
17. Hu H, Wang K, Wang L, Du Y, Chen J, Li Y, et al. He-Jie-Shen-Shi Decoction as an Adjuvant Therapy on Severe Coronavirus Disease 2019: A Retrospective Cohort and Potential Mechanistic Study. *Front Pharmacol.* 2021;**12**:700498.
18. Yang Q, Sun QG, Jiang B, Xu HJ, Luo M, Xie P, et al. Retrospective clinical study on treatment of COVID-19 patients with integrated traditional Chinese and western medicine. *Chinese Traditional and Herbal Drugs.* 2020; **51**: 2050-2054.
19. Chen L, Zhang A, Li QT, Cui Y, Yuan GD. Evaluation of clinical value of Xuebijing combined with human immunoglobulin in severe and critically ill patients with coronavirus disease 2019. *Chinese Critical Care Medicine.* 2021; **33**: 399-404.
20. Shu Z, Chang K, Zhou Y, Peng C, Li X, Cai W, et al. Add-On Chinese Medicine for Coronavirus Disease 2019 (ACCORD): A Retrospective Cohort Study of Hospital Registries. *Am J Chin Med.* 2021;**49**:543-75.
21. Zhang L, Zheng X, Bai X, Wang Q, Chen B, Wang H, et al. Association between use of Qingfei Paidu Tang and mortality in hospitalized patients with COVID-19: A national retrospective registry study. *Phytomedicine.* 2021;**85**:153531.
22. Ai Z, Zhou S, Li W, Wang M, Wang L, Hu G, et al. "Fei Yan No. 1" as a Combined Treatment for COVID-19: An Efficacy and Potential Mechanistic Study. *Front Pharmacol.* 2020;**11**:581277.
23. Hu YQ, Lu JQ, Huang J, Huang QH, Jin HH, Ma YT, *et al.* Clinical Observation of 31 cases with COVID-19 treated with Guizhi Erchen Decoction based on Triple-Jiao Sequential Therapy. *Chinese Archives of Traditional Chinese Medicine.* 2020; **38**: 1-5.
24. Lian J, Zhang ShJ, Li GL, Shang D, Wang QY, Xu LS, et al. Retrospective analysis of 38 cases with coronavirus disease 2019 treated by integrated traditional Chinese and western medicine. *Journal of Traditional Chinese Medicine.* 2020; **61**: 2126 -2130.
25. Wang LQ, Hu GM, Ba YM, He CX, Li WN, Zhang X. A retrospective study on the treatment of coronavirus disease 2019 with “Pneumonia No.1” combined with conventional western medicine. *Journal of Emergency in Traditional Chinese Medicine.* 2021; **30**: 10-12.
26. Shi J, Yang ZG, Ye C, Chen SS, Lu YF, Lv Y, et al. Clinical observation on 49 cases of non-critical COVID-19 in Shanghai treated by integrated traditional Chinese and western medicine. *Shanghai Journal of Traditional Chinese Medicine.* 2020; **54**: 30-35.
27. Xia WG, An CQ, Zheng QJ, Zhang JX, Huang M, Wang Y, *et al*. Clinical observation on 34 patients with novel coronavirus pneumonia (COVID-19) treated with integrated traditional Chinese and western medicine. *Journal of Traditional Chinese Medicine.* 2020; **61**: 375-382.
28. An YW, Yuan B, Wang JC, Wang C, Liu TT, Song S, et al. Clinical characteristics and impacts of traditional Chinese medicine treatment on the convalescents of COVID-19. *Int J Med Sci.* 2021;**18**:646-51.
