# Supplementary material for: Efficacy and mechanisms of traditional Chinese medicine for COVID-19: a systematic review
Source: Chin Med. 2022 Feb 28;17:30. doi: 10.1186/s13020-022-00587-7 (PMC8883015; doi:10.1186/s13020-022-00587-7)
Supplement: Supplementary file 5 — Additional file 5. Efficacy assessment of secondary outcomes. [file 13020_2022_587_MOESM5_ESM.docx]

Additional file 5. Efficacy assessment of secondary outcomes

| Secondary outcome | | | Number of study | Sample size | | Statistical method | I^2^ | Effect value | p‐value |
| --- | --- | --- | --- | --- | --- | --- | --- | --- | --- |
| Outcome measure | Study type | Clinical type |  | TCM | CWM |  |  |  |  |
| Total effective rate | RCTs | mild group | 8 | 490 | 491 | RR, fixed, 95% | 36% | 1.19 [1.12, 1.27] | < 0.0001 |
|  |  | severe group | 1 | 57 | 54 | / | / | 2.55 [1.52, 4.27] | 0.0004 |
|  |  | mixed group | 5 | 220 | 203 | RR, random, 95% | 60% | 1.10 [0.99, 1.22] | 0.07 |
|  |  | convalescent group | 1 | 30 | 30 | / | / | 1.42 [1.06, 1.91] | 0.02 |
|  |  | Total | 15 | 797 | 778 | RR, random, 95% | 70% | 1.18 [1.09, 1.27] | < 0.0001 |
|  | RSs | mild group | 1 | 51 | 51 | / | / | 1.26 [1.01, 1.56] | 0.04 |
| Clinical cure rate | RCTs | mild group | 2 | 80 | 79 | RR, fixed, 95% | 0% | 1.74 [1.26, 2.42] | 0.0008 |
|  |  | mixed group | 4 | 336 | 327 | RR, random, 95% | 61% | 1.15 [1.02, 1.30] | 0.03 |
|  |  | Total | 6 | 416 | 406 | RR, random, 95% | 75% | 1.26 [1.06, 1.49] | 0.008 |
|  | RSs | mild group | 1 | 100 | 100 | / | / | 1.15 [1.03, 1.29] | 0.02 |
|  |  | severe group | 1 | 51 | 52 | / | / | 1.17 [0.91, 1.50] | 0.22 |
|  |  | mixed group | 2 | 65 | 39 | RR, fixed, 95% | 1% | 1.32 [1.05, 1.65] | 0.02 |
|  |  | Total | 4 | 216 | 191 | RR, fixed, 95% | 0% | 1.19 [1.08, 1.32] | 0.0005 |
| Lung CT improvement rate | RCTs | mild group | 8 | 470 | 461 | RR, fixed, 95% | 0% | 1.14 [1.07, 1.22] | 0.0002 |
|  |  | mixed group | 2 | 190 | 188 | RR, fixed, 95% | 0% | 1.33 [1.16, 1.53] | < 0.0001 |
|  |  | convalescent group | 1 | 38 | 14 | / | / | 1.10 [0.92, 1.30] | 0.29 |
|  |  | Total | 11 | 698 | 663 | RR, fixed, 95% | 10% | 1.19 [1.12, 1.26] | < 0.0001 |
|  | RSs | mild group | 3 | 219 | 191 | RR, fixed, 95% | 0% | 1.28 [1.11, 1.48] | 0.0006 |
|  |  | mixed group | 3 | 114 | 57 | RR, random, 95% | 68% | 1.11 [0.88, 1.40] | 0.37 |
|  |  | Total | 6 | 333 | 248 | RR, random, 95% | 64% | 1.19 [1.01, 1.39] | 0.04 |
| TCM symptom scores | RCTs | mild group | 6 | 279 | 234 | SMD, random, 95%CI | 86% | -2.87 [-3.95, -1.78] | < 0.0001 |
|  |  | mixed group | 2 | 113 | 103 | SMD, random, 95%CI | 90% | -1.84 [-5.04, 1.35] | 0.26 |
|  |  | convalescent group | 1 | 30 | 30 | / | / | -7.30 [-11.85, -2.75] | 0.002 |
|  |  | Total | 9 | 422 | 367 | SMD, random, 95%CI | 89% | -2.75 [-3.75, -1.75] | < 0.0001 |
|  | RSs | mixed group | 5 | 199 | 123 | SMD, random, 95%CI | 86% | -3.81 [-6.29, -1.32] | 0.003 |
| Disappearance rate of fever | RCTs | mild group | 3 | 95 | 55 | RR, random, 95% | 91% | 1.14 [0.67, 1.94] | 0.63 |
|  |  | mixed group | 3 | 113 | 106 | RR, random, 95% | 81% | 1.09 [0.90, 1.32] | 0.36 |
|  |  | Total | 6 | 208 | 161 | RR, random, 95% | 84% | 1.11 [0.94, 1.30] | 0.22 |
|  | RSs | mild group | 3 | 90 | 83 | RR, fixed, 95% | 0% | 1.41 [1.16, 1.70] | 0.0004 |
| Disappearance time of fever | RCTs | mild group | 4 | 230 | 230 | SMD, random, 95%CI | 56% | -1.28 [-1.72, -0.83] | < 0.0001 |
|  |  | mixed group | 3 | 224 | 225 | SMD, random, 95%CI | 91% | -0.78 [-1.74, 0.18] | 0.11 |
|  |  | Total | 7 | 454 | 455 | SMD, random, 95%CI | 81% | -1.05 [-1.47, -0.63] | < 0.0001 |
|  | RSs | mild group | 4 | 108 | 87 | SMD, fixed, 95%CI | 21% | -1.56 [-1.98, -1.14] | < 0.0001 |
|  |  | severe group | 1 | 30 | 30 | / | / | -3.22 [-6.82, 0.38] | 0.08 |
|  |  | mixed group | 5 | 188 | 133 | SMD, random, 95%CI | 94% | -2.02 [-3.44, -0.60] | 0.005 |
|  |  | Total | 10 | 326 | 250 | SMD, random, 95%CI | 88% | -1.90 [-2.71, -1.08] | < 0.0001 |
| Disappearance rate of cough | RCTs | mild group | 3 | 126 | 87 | RR, random, 95% | 83% | 1.20 [0.77, 1.87] | 0.42 |
|  |  | mixed group | 3 | 126 | 118 | RR, fixed, 95% | 19% | 1.50 [1.21, 1.84] | 0.0002 |
|  |  | Total | 6 | 252 | 205 | RR, random, 95% | 75% | 1.33 [1.01, 1.74] | 0.04 |
|  | RSs | mild group | 3 | 76 | 70 | RR, fixed, 95% | 25% | 1.97 [1.34, 2.88] | 0.0005 |
| Disappearance time of cough | RCTs | mild group | 4 | 230 | 230 | SMD, random, 95%CI | 93% | -2.22 [-4.24, -0.20] | 0.03 |
|  | RSs | mild group | 3 | 163 | 144 | SMD, random, 95%CI | 90% | -0.92 [-2.41, 0.56] | 0.22 |
|  |  | severe group | 1 | 30 | 31 | / | / | -2.78 [-7.36, 1.80] | 0.23 |
|  |  | Total | 4 | 193 | 175 | SMD, random, 95%CI | 86% | -1.06 [-2.47, 0.34] | 0.14 |
| Disappearance rate of fatigue | RCTs | mild group | 3 | 102 | 66 | RR, fixed, 95% | 0% | 1.26 [1.05, 1.52] | 0.01 |
|  |  | mixed group | 2 | 73 | 68 | RR, fixed, 95% | 0% | 1.30 [1.05, 1.60] | 0.02 |
|  |  | Total | 5 | 175 | 134 | RR, fixed, 95% | 0% | 1.28 [1.11, 1.47] | 0.0005 |
|  | RSs | mild group | 3 | 61 | 61 | RR, fixed, 95% | 0% | 1.68 [1.14, 2.47] | 0.008 |
| Disappearance time of fatigue | RCTs | mild group | 2 | 140 | 140 | SMD, random, 95%CI | 93% | -1.09 [-2.62, 0.44] | 0.16 |
|  | RSs | mild group | 3 | 159 | 142 | SMD, random, 95%CI | 93% | -1.12 [-2.23, -0.00] | 0.05 |
| Discharge rate | RCTs | mild group | 2 | 57 | 56 | RR, fixed, 95% | 0% | 1.33 [0.91, 1.94] | 0.14 |
|  |  | mixed group | 1 | 100 | 100 | / | / | 0.97 [0.77, 1.22] | 0.77 |
|  |  | Total | 3 | 157 | 156 | RR, fixed, 95% | 1% | 1.07 [0.88, 1.30] | 0.51 |
|  | RSs | mild group | 2 | 104 | 68 | RR, fixed, 95% | 17% | 1.63 [1.21, 2.20] | 0.001 |
|  |  | severe group | 4 | 293 | 265 | RR, random, 95% | 67% | 1.35 [1.07, 1.71] | 0.01 |
|  |  | mixed group | 1 | 93 | 93 | / | / | 1.14 [1.03, 1.26] | 0.009 |
|  |  | Total | 7 | 490 | 426 | RR, random, 95% | 72% | 1.33 [1.11, 1.60] | 0.002 |
| Length of hospital stay | RCTs | mild group | 1 | 70 | 70 | / | / | -3.10 [-3.72, -2.48] | ＜ 0.0001 |
|  |  | mixed group | 1 | 55 | 43 | / | / | -2.51 [-4.98, -0.04] | 0.05 |
|  |  | Total | 2 | 125 | 113 | SMD, fixed, 95%CI | 0% | -3.07 [-3.67, -2.46] | ＜ 0.0001 |
|  | RSs | mild group | 2 | 101 | 64 | SMD, random, 95%CI | 67% | 0.41 [-1.69, 2.51] | 0.7 |
|  |  | severe group | 3 | 114 | 57 | SMD, fixed, 95%CI | 0% | -2.03 [-3.25, -0.80] | 0.001 |
|  |  | Total | 5 | 215 | 121 | SMD, random, 95%CI | 58% | -0.90 [-2.24, 0.44] | 0.19 |
| The rate of negative 2019-nCoV nucleic acids tests | RCTs | mild group | 2 | 62 | 58 | RR, fixed, 95% | 23% | 1.85 [1.38, 2.49] | ＜ 0.0001 |
|  |  | mixed group | 2 | 169 | 162 | RR, fixed, 95% | 0% | 1.10 [0.97, 1.25] | 0.12 |
|  |  | Total | 4 | 231 | 220 | RR, random, 95% | 72% | 1.37 [1.05, 1.79] | 0.02 |
|  | RSs | mild group | 3 | 119 | 81 | RR, fixed, 95% | 0% | 1.04 [0.89, 1.22] | 0.59 |
| The conversion time of negative 2019-nCoV nucleic acids tests | RCTs | mild group | 1 | 40 | 40 | / | / | -2.30 [-3.75, -0.85] | 0.002 |
|  |  | mixed group | 3 | 224 | 225 | SMD, fixed, 95%CI | 0% | -1.47 [-2.02, -0.91] | ＜ 0.0001 |
|  |  | Total | 4 | 264 | 265 | SMD, fixed, 95%CI | 0% | -1.58 [-2.09, -1.06] | ＜ 0.0001 |
|  | RSs | mild group | 2 | 23 | 18 | SMD, fixed, 95%CI | 0% | -2.54 [-4.86, -0.23] | 0.03 |
|  |  | severe group | 2 | 55 | 64 | SMD, fixed, 95%CI | 24% | -3.50 [-5.52, -1.48] | 0.0007 |
|  |  | Total | 4 | 78 | 82 | SMD, fixed, 95%CI | 0% | -3.09 [-4.61, -1.57] | ＜ 0.0001 |
| Incidence of adverse events | RCTs | mild group | 11 | 679 | 635 | RR, random, 95% | 61% | 0.60 [0.25, 1.46] | 0.26 |
|  |  | severe group | 1 | 57 | 54 | / | / | 0.98 [0.94, 1.03] | 0.49 |
|  |  | mixed group | 8 | 504 | 491 | RR, random, 95% | 53% | 0.84 [0.43, 1.63] | 0.6 |
|  |  | Total | 20 | 1240 | 1180 | RR, random, 95% | 67% | 0.80 [0.59, 1.08] | 0.14 |
|  | RSs | mild group | 4 | 176 | 198 | RR, fixed, 95% | 25% | 1.23 [0.57, 2.66] | 0.60 |
|  |  | severe group | 1 | 23 | 32 | / | / | 0.83 [0.22, 3.15] | 0.79 |
|  |  | mixed group | 5 | 2720 | 6471 | RR, fixed, 95% | 0% | 0.81 [0.71, 0.92] | 0.0008 |
|  |  | Total | 10 | 2919 | 6701 | RR, fixed, 95% | 0% | 0.82 [0.72, 0.92] | 0.001 |
| WBC count | RCTs | mild group | 7 | 446 | 447 | SMD, random, 95%CI | 80% | 0.27 [0.05, 0.49] | 0.02 |
|  |  | mixed group | 1 | 58 | 60 | / | / | -0.22 [-0.76, 0.32] | 0.43 |
|  |  | convalescent group | 1 | 38 | 14 | / | / | 0.87 [-0.07, 1.81] | 0.07 |
|  |  | Total | 9 | 542 | 521 | SMD, random, 95%CI | 77% | 0.25 [0.03, 0.46] | 0.03 |
|  | RSs | mild group | 4 | 295 | 324 | SMD, random, 95%CI | 78% | 0.39 [-0.13, 0.91] | 0.14 |
|  |  | severe group | 3 | 231 | 148 | SMD, fixed, 95%CI | 20% | -0.03 [-0.53, 0.48] | 0.91 |
|  |  | mixed group | 3 | 173 | 149 | SMD, fixed, 95%CI | 0% | -0.04 [-0.53, 0.45] | 0.86 |
|  |  | convalescent group | 1 | 143 | 425 | / | / | -0.33 [-0.55, -0.11] | 0.003 |
|  |  | Total | 11 | 842 | 1046 | SMD, random, 95%CI | 88% | 0.14 [-0.30, 0.58] | 0.54 |
| LYM count | RCTs | mild group | 5 | 276 | 277 | SMD, random, 95%CI | 98% | 0.31 [0.09, 0.53] | 0.005 |
|  |  | mixed group | 2 | 113 | 103 | SMD, random, 95%CI | 66% | 0.25 [-0.10, 0.59] | 0.16 |
|  |  | convalescent group | 1 | 38 | 14 | / | / | -0.25 [-0.49, -0.01] | 0.05 |
|  |  | Total | 8 | 427 | 394 | SMD, random, 95%CI | 97% | 0.23 [0.05, 0.41] | 0.01 |
|  | RSs | mild group | 2 | 158 | 198 | SMD, fixed, 95%CI | 0% | 0.14 [0.03, 0.24] | 0.009 |
|  |  | severe group | 2 | 74 | 84 | SMD, fixed, 95%CI | 0% | 0.09 [0.06, 0.12] | ＜ 0.0001 |
|  |  | mixed group | 2 | 142 | 128 | SMD, random, 95%CI | 91% | 0.25 [-0.63, 1.13] | 0.57 |
|  |  | convalescent group | 1 | 143 | 425 | / | / | -0.11 [-0.20, -0.02] | 0.01 |
|  |  | Total | 7 | 517 | 835 | SMD, random, 95%CI | 82% | 0.10 [-0.01, 0.21] | 0.25 |
| LYM% | RCTs | mild group | 4 | 239 | 239 | SMD, random, 95%CI | 93% | 2.12 [-0.36, 4.60] | 0.09 |
|  | RSs | mild group | 1 | 100 | 100 | / | / | 3.60 [3.14, 4.06] | ＜ 0.0001 |
|  |  | severe group | 3 | 96 | 104 | SMD, random, 95%CI | 91% | -0.28 [-1.44, 0.88] | 0.63 |
|  |  | mixed group | 1 | 31 | 21 | / | / | 2.25 [0.23, 4.27] | 0.03 |
|  |  | Total | 5 | 227 | 225 | SMD, random, 95%CI | 98% | 0.49 [-1.39, 2.38] | 0.61 |
| CRP | RCTs | mild group | 8 | 408 | 405 | SMD, random, 95%CI | 87% | -4.98 [-6.62, -3.34] | ＜ 0.0001 |
|  |  | mixed group | 3 | 149 | 149 | SMD, random, 95%CI | 99% | -15.30 [-27.12, -3.48] | 0.01 |
|  |  | Total | 11 | 557 | 554 | SMD, random, 95%CI | 97% | -7.65 [-10.22, -5.08] | ＜ 0.0001 |
|  | RSs | mild group | 4 | 294 | 322 | SMD, random, 95%CI | 93% | -1.56 [-3.98, 0.87] | 0.21 |
|  |  | severe group | 2 | 237 | 148 | SMD, random, 95%CI | 99% | -45.72 [-98.53, 7.08] | 0.09 |
|  |  | mixed group | 2 | 80 | 56 | SMD, random, 95%CI | 88% | -5.74 [-8.87, -2.60] | 0.0003 |
|  |  | convalescent group | 1 | 143 | 425 | / | / | 0.16 [0.04, 0.28] | 0.01 |
|  |  | Total | 9 | 754 | 951 | SMD, random, 95%CI | 99% | -12.03 [-15.90, -8.16] | ＜ 0.0001 |
| IL-6 | RCTs | mild group | 2 | 56 | 55 | SMD, fixed, 95%CI | 0% | -0.39 [-0.81, 0.04] | 0.07 |
|  |  | mixed group | 2 | 91 | 89 | SMD, random, 95%CI | 84% | -11.78 [-21.68, -1.88] | 0.02 |
|  |  | Total | 4 | 147 | 144 | SMD, random, 95%CI | 96% | -4.81 [-9.33, -0.29] | 0.04 |
|  | RSs | mild group | 2 | 167 | 210 | SMD, fixed, 95%CI | 0% | -1.56 [-3.98, 0.87] | 0.21 |
|  |  | severe group | 1 | 23 | 32 | / | / | 0.56 [-1.64, 2.76] | 0.62 |
|  |  | convalescent group | 1 | 143 | 425 | / | / | -13.84 [-19.73, -7.95] | ＜ 0.0001 |
|  |  | Total | 4 | 333 | 667 | SMD, random, 95%CI | 85% | -0.60 [-0.77, -0.44] | ＜ 0.0001 |
