# Supplementary material for: Efficacy and mechanisms of traditional Chinese medicine for COVID-19: a systematic review
Source: Chin Med. 2022 Feb 28;17:30. doi: 10.1186/s13020-022-00587-7 (PMC8883015; doi:10.1186/s13020-022-00587-7)
Supplement: Supplementary file 6 — Additional file 6. The frequency of the Chinese medicinal herbs. [file 13020_2022_587_MOESM6_ESM.docx]

Additional file 6. The frequency of the Chinese medicinal herbs

| NO | Chinese medicinal herbs | 中药名称 | Frequency |
| --- | --- | --- | --- |
| 1 | Glycyrrhizae Radix et Rhizoma | 甘草 | 39 |
| 2 | Armeniacae Semen Amarum | 苦杏仁 | 31 |
| 3 | Ephedrae Herba | 麻黄 | 27 |
| 4 | Gypsum Fibrosum | 石膏 | 27 |
| 5 | Poria | 茯苓 | 25 |
| 6 | Forsythiae Fructus | 连翘 | 25 |
| 7 | Scutellariae Radix | 黄芩 | 24 |
| 8 | Pinelliae Rhizoma Praeparatum | 法半夏 | 22 |
| 9 | Pogostemonis Herba | 广藿香 | 22 |
| 10 | Lonicerae Japonicae Flos | 金银花 | 22 |
| 11 | Bupleuri Radix | 柴胡 | 20 |
| 12 | Citri Reticulatae Pericarpium | 陈皮 | 19 |
| 13 | Atractylodis Macrocephalae Rhizoma | 白术 | 18 |
| 14 | Rhei Radix et Rhizoma | 大黄 | 17 |
| 15 | Magnoliae Officinalis Cortex | 厚朴 | 14 |
| 16 | Isatidis Radix | 板蓝根 | 13 |
| 17 | Tsaoko Fructus | 草果 | 12 |
| 18 | Menthae Haplocalycis Herba | 薄荷 | 11 |
| 19 | Zingiberis Rhizoma Recens | 生姜 | 11 |
| 20 | Dryopteridis Crassirhizomatis Rhizoma | 绵马贯众 | 11 |
| 21 | Atractylodis Rhizoma | 苍术 | 10 |
| 22 | Houttuyniae Herba | 鱼腥草 | 10 |
| 23 | Platycodonis Radix | 桔梗 | 10 |
| 24 | Astragali Radix | 黄芪 | 10 |
| 25 | Rhodiolae Crenulatae Radix et Rhizoma | 红景天 | 10 |
| 26 | Codonopsis Radix | 党参 | 9 |
| 27 | Descurainiae Semen | 葶苈子 | 9 |
| 28 | Polygoni Cuspidati Rhizoma et Radix | 虎杖 | 9 |
| 29 | Arecae Semen | 槟榔 | 9 |
| 30 | Artemisiae Annuae Herba | 青蒿 | 8 |
| 31 | Cinnamomi Ramulus | 桂枝 | 8 |
| 32 | Ginseng Radix et Rhizoma | 人参 | 8 |
| 33 | Cicadae Periostracum | 蝉蜕 | 8 |
| 34 | Salviae Miltiorrhizae Radix et Rhizoma | 丹参 | 7 |
| 35 | Anemarrhenae Rhizoma | 知母 | 7 |
| 36 | Fritillariae Thunbrgii Bulbus | 浙贝母 | 7 |
| 37 | Farfarae Flos | 款冬花 | 7 |
| 38 | Asteris Radix et Rhizoma | 紫菀 | 7 |
| 39 | Paeoniae Radix Rubra | 赤芍 | 7 |
| 40 | Scrophulariae Radix | 玄参 | 6 |
| 41 | Belamcandae Rhizome | 射干 | 6 |
| 42 | Dioscoreae Rhizoma | 山药 | 6 |
| 43 | Coicis Semen | 薏苡仁 | 6 |
| 44 | Peucedani Radix | 前胡 | 5 |
| 45 | Fritiliariae Cirrhosae Bulbus | 川贝母 | 5 |
| 46 | Schisandrae Chinensis Fructus | 五味子 | 5 |
| 47 | Alismatis Rhizoma | 泽泻 | 5 |
| 48 | Verbenae Herba | 马鞭草 | 5 |
| 49 | Trichosanthis Fructus | 瓜蒌 | 5 |
| 50 | Aconiti Lateralis Radix Praeparata | 附子 | 5 |
| 51 | Ophiopogonis Radix | 麦冬 | 5 |
| 52 | Herba Patriniae | 败酱草 | 5 |
| 53 | Pseudostellariae Radix | 太子参 | 4 |
| 54 | Arctii Fructus | 牛蒡子 | 4 |
| 55 | Asari Radix et Rhizoma | 细辛 | 4 |
| 56 | Aurantii Fructus Immaturus | 枳实 | 4 |
| 57 | Hordei Fructus Germinatus | 麦芽 | 4 |
| 58 | Isatidis Folium | 大青叶 | 4 |
| 59 | Angelicae Sinensis Radix | 当归 | 4 |
| 60 | Amomi Fructus Rotundus | 豆蔻 | 4 |
| 61 | Amomi Fructus | 砂仁 | 4 |
| 62 | Polyporus | 猪苓 | 4 |
| 63 | Cremastrae Pseudobulbus | 山慈菇 | 3 |
| 64 | Mume Fructus | 乌梅 | 3 |
| 65 | Pheretima | 地龙 | 3 |
| 66 | Talci Pulvis | 滑石粉 | 3 |
| 67 | Rehmanniae Radix | 地黄 | 3 |
| 68 | Notopterygii Rhizoma et Radix | 羌活 | 3 |
| 69 | Eupatorii Herba | 佩兰 | 3 |
| 70 | Persicae Semen | 桃仁 | 3 |
| 71 | Carthami Flos | 红花 | 3 |
| 72 | Chuanxiong Rhizoma | 川芎 | 3 |
| 73 | Taraxacl Herba | 蒲公英 | 3 |
| 74 | Crataegi Fructs | 山楂 | 3 |
| 75 | Zingiberis Rhizoma | 干姜 | 2 |
| 76 | Perillae Folium | 紫苏叶 | 2 |
| 77 | Bombyx Batryticatus | 僵蚕 | 2 |
| 78 | Curcumae Longae Rhizoma | 姜黄 | 2 |
| 79 | Lilii Bulbus | 百合 | 2 |
| 80 | Lablab Semen Album | 白扁豆 | 2 |
| 81 | Bubali Cornu | 水牛角 | 2 |
| 82 | Cynanchi Paniculati Radix et Rhizoma | 徐长卿 | 2 |
| 83 | Coptidis Rhizoma | 黄连 | 2 |
| 84 | Lophatheri Herba | 淡竹叶 | 2 |
| 85 | Mori Follum | 桑叶 | 2 |
| 86 | Andrographis Herba | 穿心莲 | 2 |
| 87 | Tetrapanacis Medulla | 通草 | 2 |
| 88 | Paeoniae Radix Alba | 白芍 | 2 |
| 89 | Acori Tataninowii Rhizoma | 石菖蒲 | 2 |
| 90 | Angelicae Dahuricae Radix | 白芷 | 2 |
| 91 | Artemisiae Scopariae Herba | 茵陈 | 2 |
| 92 | Sargentodoxae Caulis | 大血藤 | 2 |
| 93 | Hirudo | 水蛭 | 2 |
| 94 | Arecae Pericarpium | 大腹皮 | 2 |
| 95 | Gardeniae Fructus | 栀子 | 2 |
| 96 | Medicated leaven | 神曲 | 2 |
| 97 | Goral Horn | 山羊角 | 2 |
| 98 | Jujubae Fructus | 大枣 | 2 |
| 99 | Mori Cortex | 桑白皮 | 2 |
