# Supplementary material for: Efficacy and mechanisms of traditional Chinese medicine for COVID-19: a systematic review
Source: Chin Med. 2022 Feb 28;17:30. doi: 10.1186/s13020-022-00587-7 (PMC8883015; doi:10.1186/s13020-022-00587-7)
Supplement: Supplementary file 7 — Additional file 7. Antiviral components and targets. [file 13020_2022_587_MOESM7_ESM.docx]

Additional file 7. Antiviral components and targets

| Component | PubChem CID | Targets | References | Chinese medicinal herbs |
| --- | --- | --- | --- | --- |
| (-)-Medicocarpin | 23724664 | ACE2, 3CLpro, Spro | Li XL, et al. [80] | Glycyrrhizae Radix et Rhizoma (甘草) |
| (+)-Catechin | 9064 | ACE2, 3CLpro, Spro | Li XL, et al. [80] | Armeniacae Semen Amarum (苦杏仁)，Ephedra Herba (麻黄)，Polygoni Cuspidati Rhizoma et Radix (虎杖)，Cinnamomi Ramulus (桂枝)，Paeoniae Radix Rubra (赤芍)，Hordei Fructus Germinatus (麦芽)，Perillae Folium (紫苏叶)，Paeoniae Radix Alba (白芍)，Jujubae Fructus (大枣) |
| (+)-Leucocyanidin | 155206 | ACE2, 3CLpro, Spro | Li XL, et al. [80] | Ephedra Herba (麻黄) |
| (2R)-7-hydroxy-2-(4-hydroxyphenyl)chroman-4-one | 928837 | ACE2, 3CLpro, Spro | Li XL, et al. [80] | Glycyrrhizae Radix et Rhizoma (甘草) |
| (2S)-6-(2,4-dihydroxyphenyl)-2-(2-hydroxypropan-2-yl)-4-methoxy-2,3-dihydrofuro[3,2-g]chromen-7-one | 637112 | ACE2, 3CLpro, Spro | Li XL, et al. [80] | Glycyrrhizae Radix et Rhizoma (甘草) |
| 1,3-dihydroxy-8,9-dimethoxy-6-benzofurano[3,2-c]chromenone | 11602329 | ACE2, 3CLpro, Spro | Li XL, et al. [80] | Glycyrrhizae Radix et Rhizoma (甘草) |
| 1-Methoxyphaseollidin | 480873 | ACE2, 3CLpro, Spro | Li XL, et al. [80] | Glycyrrhizae Radix et Rhizoma (甘草) |
| 2-(3,4-dihydroxyphenyl)-5,7-dihydroxy-6-(3-methylbut-2-enyl)chromone | 14604081 | ACE2, 3CLpro, Spro | Li XL, et al. [80] | Glycyrrhizae Radix et Rhizoma (甘草) |
| 24-Ethylcholest-4-en-3-one | 15596633 | ACE2, 3CLpro | Li XL, et al. [80] | Ephedra Herba (麻黄)，Pinelliae Rhizoma Praeparatum (法半夏)，Isatidis Radix (板蓝根)，Amomi Fructus (砂仁) |
| 3'-Hydroxy-4'-O-Methylglabridin | 15228662 | ACE2, 3CLpro, Spro | Li XL, et al. [80] | Glycyrrhizae Radix et Rhizoma (甘草) |
| 3'-Methoxyglabridin | 5319439 | ACE2, 3CLpro, Spro | Li XL, et al. [80] | Glycyrrhizae Radix et Rhizoma (甘草) |
| 5-Prenylbutein | 11267805 | ACE2, 3CLpro, Spro | Li XL, et al. [80] | Glycyrrhizae Radix et Rhizoma (甘草) |
| 7,2',4'-trihydroxy－5-methoxy-3－arylcoumarin | 25015742 | ACE2, 3CLpro, Spro | Li XL, et al. [80] | Glycyrrhizae Radix et Rhizoma (甘草) |
| 7-Acetoxy-2-methylisoflavone | 268208 | ACE2, 3CLpro, Spro | Li XL, et al. [80] | Glycyrrhizae Radix et Rhizoma (甘草) |
| 7-Methoxy-2-methyl isoflavone | 354368 | ACE2, 3CLpro, Spro | Li XL, et al. [80] | Glycyrrhizae Radix et Rhizoma (甘草)，Codonopsis Radix (党参) |
| Acetoside | 5281800 | 3CLpro | Ren X, et al. [89] | Rehmanniae Radix (地黄) |
| Amygdalin | 656516 | ACE2, 3CLpro, Rdrp | Li Y, et al. [86] | Armeniacae Semen Amarum (苦杏仁)，Mume Fructus (乌梅)，Persicae Semen (桃仁) |
| Anhydrosafflor yellow B | 102240413 | ACE2, 3CLpro, Spro | Xing Y, et al. [90] | Carthami Flos (红花) |
| Apigenin | 5280443 | ACE2, 3CLpro, Spro | Wang H, et al. [82] Xing Y, et al. [90] | Ephedra Herba (麻黄)，Scutellariae Radix (黄芩)，Pogostemonis Herba (广藿香)，Lonicerae Japonicae Flos (金银花)，Isatidis Radix (板蓝根)，Menthae Haplocalycis Herba (薄荷)，Codonopsis Radix (党参)，Polygoni Cuspidati Rhizoma et Radix (虎杖)，Artemisiae Annuae Herba (青蒿)，Salviae Miltiorrhizae Radix et Rhizoma (丹参)，Asteris Radix et Rhizoma (紫菀)，Belamcandae Rhizome (射干)，Verbenae Herb (马鞭草)，Herba Patriniae (败酱草)，Aurantii Fructus Immaturus (枳实)，Carthami Flos (红花)，Perillae Folium (紫苏叶)，Acori Tataninowii Rhizoma (石菖蒲） |
| Artemetin | 5320351 | ACE2, 3CLpro, Spro, Rdrp | Ye MB, et al. [79] | Artemisiae Annuae Herba (青蒿)，Verbenae Herb (马鞭草) |
| Astragaloside IV | 13943297 | ACE2, 3CLpro, Spro, Rdrp | Ge CL, et al. [77] Ye MB, et al. [79] | Astragali Radix (黄芪) |
| Baicalein | 5281605 | ACE2, 3CLpro, Spro, Rdrp | Tao QY, et al. [72] Ye MB, et al. [79] | Scutellariae Radix (黄芩)，Pinelliae Rhizoma Praeparatum (法半夏)，Paeoniae Radix Rubra (赤芍)，Carthami Flos (红花) |
| Baicalin | 64982 | ACE2, 3CLpro, Spro | Li XL, et al. [80] | Pinelliae Rhizoma Praeparatum (法半夏)，Bupleuri Radix (柴胡)，Salviae Miltiorrhizae Radix et Rhizoma (丹参)，Paeoniae Radix Rubra (赤芍)，Carthami Flos (红花) |
| Beta-carotene | 5280489 | ACE2, 3CLpro, Spro, Rdrp | Ye MB, et al. [79] | Carthami Flos (红花)，Lablab Semen Album (白扁豆)，Jujubae Fructus (大枣) |
| beta-Sitosterol | 222284 | ACE2, 3CLpro, Rdrp, Spro | Tao QY, et al. [72] Li XL, et al. [78] Ye MB, et al. [79] | Ephedra Herba (麻黄)，Forsythiae Fructus (连翘)，Scutellariae Radix (黄芩)，Pinelliae Rhizoma Praeparatum (法半夏)，Lonicerae Japonicae Flos (金银花)，Rhei Radix et Rhizoma (大黄），Isatidis Radix (板蓝根)，Zingiber Officinale Roscoe (生姜)，Descurainiae Semen (葶苈子)，Polygoni Cuspidati Rhizoma et Radix (虎杖)，Cinnamomi Ramulus (桂枝)，Ginseng Radix et Rhizoma (人参)，Fritillariae Thunbrgii Bulbus (浙贝母)，Farfarae Flos (款冬花)，Asteris Radix et Rhizoma (紫菀)，Paeoniae Radix Rubra (赤芍)，Scrophulariae Radix (玄参)，Peucedani Radix (前胡)，Fritiliariae Cirrhosae Bulbus (川贝母)，Verbenae Herb (马鞭草)，Herba Patriniae (败酱草)，Pseudostellariae Radix (太子参)，Arctii Fructus (牛蒡子)，Isatidis Folium (大青叶)，Angelicae Sinensis Radix (当归)，Amomi Fructus (砂仁)，Cremastrae Pseudobulbus (山慈菇)，Mume Fructus (乌梅)，Rehmanniae Radix (地黄)，Persicae Semen (桃仁)，Carthami Flos (红花)，Taraxacl Herba (蒲公英)，Perillae Folium (紫苏叶)，Lilii Bulbus (百合)，Mori Follum (桑叶)，Paeoniae Radix Alba (白芍)，Angelicae Dahuricae Radix (白芷)，Artemisiae Scopariae Herba (茵陈)，Sargentodoxae Caulis (大血藤)，Gardeniae Fructus (栀子)，Mori Cortex (桑白皮) |
| Bicuculline | 10237 | ACE2, 3CLpro, Spro, Rdrp | Gao LQ, et al. [76] Ye MB, et al. [79] | Forsythiae Fructus (连翘) |
| Calycosin | 5280448 | ACE2, 3CLpro, Spro, Rdrp | Ye MB, et al. [79] Li XL, et al. [80] | Glycyrrhizae Radix et Rhizoma (甘草)，Astragali Radix (黄芪) |
| Campest-5-en-3beta-ol | 173183 | ACE2, 3CLpro, Spro | Li XL, et al. [80] | Ephedra Herba (麻黄)，Paeoniae Radix Rubra (赤芍)，Mume Fructus (乌梅) |
| Chryseriol | 5280666 | 3CLpro, Rdrp | Ye MB, et al. [79] | Lonicerae Japonicae Flos (金银花) |
| Cirsiliol | 160237 | 3CLpro, Rdrp | Ye MB, et al. [79] | Artemisiae Annuae Herba (青蒿) |
| Delphinidin | 68245 | ACE2, 3CLpro, Spro | Li XL, et al. [80] | Ephedra Herba (麻黄)，Hordei Fructus Germinatus (麦芽) |
| Diosgenin | 99474 | ACE2, 3CLpro | Mu CL, et al. [75] | Anemarrhenae Rhizoma (知母)，Rhizoma Dioscoreae (山药) |
| Diosmetin | 5281612 | ACE2, 3CLpro, Spro | Li XL, et al. [80] | Ephedra Herba (麻黄)，Menthae Haplocalycis Herba (薄荷)，Verbenae Herb (马鞭草)，Trichosanthis Fructus (瓜蒌)，Notopterygii Rhizoma et Radix (羌活) |
| Ellagic acid | 5281855 | RdRp, Nsp14 | Cai Y, et al. [83] | Paeoniae Radix Rubra (赤芍) |
| Emodin | 3220 | ACE2, 3CLpro | Du HX, et al. [87] | Rhei Radix et Rhizoma (大黄），Isatidis Radix (板蓝根)，Menthae Haplocalycis Herba (薄荷)，Lilii Bulbus (百合)，Sargentodoxae Caulis (大血藤) |
| Eriodictyol | 440735 | ACE2, 3CLpro, Spro | Li XL, et al. [80] | Ephedra Herba (麻黄)，Menthae Haplocalycis Herba (薄荷) |
| Eriodyctiol (flavanone) | 373261 | 3CLpro, Plpro | Chen J, et al. [74] | Scutellariae Radix (黄芩)，Lonicerae Japonicae Flos (金银花)，Aurantii Fructus Immaturus (枳实) |
| Estrone | 5870 | ACE2, 3CLpro | Gao K, et al. [93] | Armeniacae Semen Amarum (苦杏仁) |
| Eurycarpin A | 5317300 | ACE2, 3CLpro, Spro | Li XL, et al. [80] | Glycyrrhizae Radix et Rhizoma (甘草) |
| Formononetin | 5280378 | ACE2, 3CLpro, Spro, Rdrp | Tao QY, et al. [72] Ye MB, et al. [79] Li XL, et al. [80] | Glycyrrhizae Radix et Rhizoma (甘草)，Astragali Radix (黄芪) |
| Fumarine | 4970 | 3CLpro, Rdrp, Spro | Li XL, et al. [78] | Ginseng Radix et Rhizoma (人参) |
| Gadelaidic acid | 5460988 | ACE2, 3CLpro, Spro | Li XL, et al. [80] | Glycyrrhizae Radix et Rhizoma (甘草) |
| Gancaonin A | 5317478 | ACE2, 3CLpro, Spro | Li XL, et al. [80] | Glycyrrhizae Radix et Rhizoma (甘草) |
| Gancaonin B | 5317479 | ACE2, 3CLpro, Spro | Li XL, et al. [80] | Glycyrrhizae Radix et Rhizoma (甘草) |
| Gancaonin G | 480780 | ACE2, 3CLpro | Li XL, et al. [80] | Glycyrrhizae Radix et Rhizoma (甘草) |
| Gancaonin H | 5481949 | ACE2, 3CLpro, Spro | Li XL, et al. [80] | Glycyrrhizae Radix et Rhizoma (甘草) |
| Gancaonin L | 14604077 | ACE2, 3CLpro, Spro | Li XL, et al. [80] | Glycyrrhizae Radix et Rhizoma (甘草) |
| Gancaonin M | 14604078 | ACE2, 3CLpro, Spro | Li XL, et al. [80] | Glycyrrhizae Radix et Rhizoma (甘草) |
| Genkwanin | 5281617 | ACE2, 3CLpro, Spro | Li XL, et al. [80] Gao K, et al. [93] | Ephedra Herba (麻黄)，Pogostemonis Herba (广藿香)，Menthae Haplocalycis Herba (薄荷)，Artemisiae Scopariae Herba (茵陈) |
| Glabranin | 124049 | ACE2, 3CLpro, Spro | Li XL, et al. [80] | Glycyrrhizae Radix et Rhizoma (甘草) |
| Glabrene | 480774 | ACE2, 3CLpro, Spro | Li XL, et al. [80] | Glycyrrhizae Radix et Rhizoma (甘草) |
| Glabridin | 124052 | ACE2, 3CLpro, Spro | Li XL, et al. [80] Gao K, et al. [93] | Glycyrrhizae Radix et Rhizoma (甘草)，Armeniacae Semen Amarum (苦杏仁) |
| Glabrone | 5317652 | ACE2, 3CLpro, Spro | Li XL, et al. [80] | Glycyrrhizae Radix et Rhizoma (甘草)，Mori Cortex (桑白皮) |
| Glepidotin A | 5281619 | ACE2, 3CLpro, Spro | Li XL, et al. [80] | Glycyrrhizae Radix et Rhizoma (甘草) |
| Glepidotin B | 442411 | ACE2, 3CLpro, Spro | Li XL, et al. [80] | Glycyrrhizae Radix et Rhizoma (甘草) |
| Glyasperin B | 480784 | ACE2, 3CLpro, Spro | Li XL, et al. [80] | Glycyrrhizae Radix et Rhizoma (甘草) |
| Glyasperin C | 480859 | ACE2, 3CLpro, Spro | Li XL, et al. [80] | Glycyrrhizae Radix et Rhizoma (甘草) |
| Glyasperin F | 392442 | ACE2, 3CLpro, Spro | Li XL, et al. [80] Ren X, et al. [89] | Glycyrrhizae Radix et Rhizoma (甘草) |
| Glycyrin | 480787 | ACE2, 3CLpro, Spro | Li XL, et al. [80] | Glycyrrhizae Radix et Rhizoma (甘草) |
| Glycyrol | 5320083 | ACE2, 3CLpro, Spro | Li XL, et al. [80] Gao K, et al. [93] | Glycyrrhizae Radix et Rhizoma (甘草)，Armeniacae Semen Amarum (苦杏仁)，Isatidis Folium (大青叶) |
| Glycyroside | 101939210 | ACE2, 3CLpro, Spro | Li XL, et al. [80] | Glycyrrhizae Radix et Rhizoma (甘草) |
| Glycyrrhiza flavonol A | 5317765 | ACE2, 3CLpro, Spro | Li XL, et al. [80] | Glycyrrhizae Radix et Rhizoma (甘草) |
| Glypallichalcone | 5317768 | ACE2, 3CLpro, Spro | Li XL, et al. [80] | Glycyrrhizae Radix et Rhizoma (甘草) |
| Glyuranolide | 195396 | ACE2, 3CLpro, Spro | Li XL, et al. [80] | Glycyrrhizae Radix et Rhizoma (甘草) |
| Glyzaglabrin | 5317777 | ACE2, 3CLpro, Spro | Li XL, et al. [80] | Glycyrrhizae Radix et Rhizoma (甘草) |
| Hederagenin | 73299 | 3CLpro, Rdrp | Ye MB, et al. [79] | Poria (茯苓)，Astragali Radix (黄芪)，Descurainiae Semen (葶苈子)，Persicae Semen (桃仁) |
| Hedysarimcoumestan B | 11558452 | ACE2, 3CLpro, Spro | Li XL, et al. [80] | Glycyrrhizae Radix et Rhizoma (甘草) |
| Herbacetin | 5280544 | ACE2, 3CLpro, Spro | Li XL, et al. [80] | Ephedra Herba (麻黄) |
| Hesperidin | 10621 | 3CLpro | Ren X, et al. [89] | Ephedra Herba (麻黄)，Citri Reticulatae Pericarpium (陈皮)，Asteris Radix et Rhizoma (紫菀)，Peucedani Radix (前胡)，Aurantii Fructus Immaturus (枳实) |
| Icos-5-enoic acid | 3349565 | ACE2, 3CLpro, Spro | Li XL, et al. [80] | Glycyrrhizae Radix et Rhizoma (甘草) |
| Indirubin | 10177 | ACE2, 3CLpro, Spro, Rdrp | Ye MB, et al. [79] | Isatidis Folium (大青叶) |
| Inermine | 91510 | ACE2, 3CLpro, Spro | Li XL, et al. [80] | Glycyrrhizae Radix et Rhizoma (甘草) |
| Inflacoumarin A | 5318437 | ACE2, 3CLpro, Spro | Li XL, et al. [80] | Glycyrrhizae Radix et Rhizoma (甘草) |
| Irisolidone | 5281781 | ACE2, 3CLpro | Gao LQ, et al. [76] | Pogostemonis Herba (广藿香) |
| Isobavachin | 193679 | ACE2, 3CLpro, Spro | Li XL, et al. [80] | Glycyrrhizae Radix et Rhizoma (甘草) |
| Isoformononetin | 3764 | ACE2, 3CLpro, Spro | Li XL, et al. [80] | Glycyrrhizae Radix et Rhizoma (甘草) |
| Isoglycyrol | 124050 | ACE2, 3CLpro, Spro | Li XL, et al. [80] | Glycyrrhizae Radix et Rhizoma (甘草) |
| Isolicoflavonol | 5318585 | ACE2, 3CLpro, Spro | Li XL, et al. [80] Ren X, et al. [89] | Glycyrrhizae Radix et Rhizoma (甘草) |
| isoquercitrin | 5280804 | ACE2, 3CLpro, Spro, Rdrp | Ye MB, et al. [79] | Bupleuri Radix (柴胡)，Tsaoko Fructus (草果)，Herba Patriniae (败酱草) |
| Isorhamnetin | 5281654 | ACE2, 3CLpro, Spro, Rdrp | Tao QY, et al. [72] Gao LQ, et al. [76] Ye MB, et al. [79] Li XL, et al. [80] Ren X, et al. [89] | Glycyrrhizae Radix et Rhizoma (甘草)，Bupleuri Radix (柴胡)，Astragali Radix (黄芪)，Descurainiae Semen (葶苈子)，Artemisiae Annuae Herba (青蒿)，Asteris Radix et Rhizoma (紫菀)，Belamcandae Rhizome (射干)，Artemisiae Scopariae Herba (茵陈) |
| Isotrifoliol | 5318679 | ACE2, 3CLpro, Spro | Li XL, et al. [80] | Glycyrrhizae Radix et Rhizoma (甘草) |
| Isovitexin | 162350 | ACE2, 3CLpro | Wang J, et al. [84] | Isatidis Radix (板蓝根)，Belamcandae Rhizome (射干)，Herba Patriniae (败酱草)，Hordei Fructus Germinatus (麦芽)，Isatidis Folium (大青叶) |
| Jaranol | 5318869 | ACE2, 3CLpro, Spro | Li XL, et al. [80] | Glycyrrhizae Radix et Rhizoma (甘草)，Astragali Radix (黄芪) |
| kaempferol | 5280863 | ACE2, 3CLpro, Spro, Rdrp | Tao QY, et al. [72] Li XL, et al. [78] Ye MB, et al. [79] Wang H, et al. [82] Gao K, et al. [93] | Glycyrrhizae Radix et Rhizoma (甘草)，Ephedra Herba (麻黄)，Forsythiae Fructus (连翘)，Lonicerae Japonicae Flos (金银花)，Bupleuri Radix (柴胡)，Dryopteridis Crassirhizomatis Rhizoma (绵马贯众)，Houttuyniae Herba (鱼腥草)，Astragali Radix (黄芪)，Rhodiolae Crenulatae Radix et Rhizoma (红景天)，Descurainiae Semen (葶苈子)，Artemisiae Annuae Herba (青蒿)，Ginseng Radix et Rhizoma (人参)，Anemarrhenae Rhizoma (知母)，Farfarae Flos (款冬花)，Asteris Radix et Rhizoma (紫菀)，Verbenae Herb (马鞭草)，Herba Patriniae (败酱草)，Arctii Fructus (牛蒡子)，Asari Radix et Rhizoma (细辛)，Mume Fructus (乌梅)，Carthami Flos (红花)，Mori Follum (桑叶)，Paeoniae Radix Alba (白芍)，Gardeniae Fructus (栀子)，Mori Cortex (桑白皮) |
| Kanzonol B | 10881804 | ACE2, 3CLpro, Spro | Li XL, et al. [80] | Glycyrrhizae Radix et Rhizoma (甘草) |
| Kanzonol F | 101666840 | ACE2, 3CLpro | Li XL, et al. [80] | Glycyrrhizae Radix et Rhizoma (甘草) |
| kanzonols W | 15380912 | ACE2, 3CLpro, Spro | Li XL, et al. [80] | Glycyrrhizae Radix et Rhizoma (甘草) |
| Leucopelargonidin | 3286789 | ACE2, 3CLpro, Spro | Li XL, et al. [80] | Ephedra Herba (麻黄) |
| Licoagrocarpin | 15840593 | 3CLpro, Spro | Li XL, et al. [80] Ren X, et al. [89] | Glycyrrhizae Radix et Rhizoma (甘草) |
| Licoagroisoflavone | 636883 | ACE2, 3CLpro, Spro | Li XL, et al. [80] | Glycyrrhizae Radix et Rhizoma (甘草) |
| Licoarylcoumarin | 10090416 | ACE2, 3CLpro, Spro | Li XL, et al. [80] | Glycyrrhizae Radix et Rhizoma (甘草) |
| Licochalcone a | 5318998 | ACE2, 3CLpro, Spro | Li XL, et al. [80] | Glycyrrhizae Radix et Rhizoma (甘草) |
| Licochalcone B | 5318999 | ACE2, 3CLpro, Spro | Li XL, et al. [80] Gao K, et al. [93] | Glycyrrhizae Radix et Rhizoma (甘草)，Armeniacae Semen Amarum (苦杏仁) |
| Licochalcone G | 49856081 | ACE2, 3CLpro, Spro | Li XL, et al. [80] Ren X, et al. [89] | Glycyrrhizae Radix et Rhizoma (甘草) |
| Licocoumarone | 503731 | ACE2, 3CLpro, Spro | Li XL, et al. [80] | Glycyrrhizae Radix et Rhizoma (甘草) |
| Licoisoflavone | 5481234 | ACE2, 3CLpro, Spro | Li XL, et al. [80] | Glycyrrhizae Radix et Rhizoma (甘草) |
| Licoisoflavone B | 5281789 | ACE2, 3CLpro, Spro | Li XL, et al. [80] | Glycyrrhizae Radix et Rhizoma (甘草) |
| Licopyranocoumarin | 122851 | ACE2, 3CLpro, Spro | Li XL, et al. [80] | Glycyrrhizae Radix et Rhizoma (甘草) |
| Licorice glycoside E | 42607811 | ACE2, 3CLpro, Spro | Li XL, et al. [80] | Glycyrrhizae Radix et Rhizoma (甘草) |
| Licoricone | 5319013 | ACE2, 3CLpro, Spro | Li XL, et al. [80] | Glycyrrhizae Radix et Rhizoma (甘草) |
| Liquiritigenin | 114829 | ACE2, 3CLpro, Spro | Li XL, et al. [80] | Glycyrrhizae Radix et Rhizoma (甘草)，Isatidis Radix (板蓝根) |
| Liquiritin | 503737 | ACE2, 3CLpro, Spro | Li XL, et al. [80] Wu HY, et al. [81] Ren X, et al. [89] | Glycyrrhizae Radix et Rhizoma (甘草)，Armeniacae Semen Amarum (苦杏仁) |
| l-stepholidine | 6917970 | ACE2, 3CLpro | Gao K, et al. [93] | Armeniacae Semen Amarum (苦杏仁) |
| Lupiwighteone | 5317480 | ACE2, 3CLpro, Spro | Li XL, et al. [80] | Glycyrrhizae Radix et Rhizoma (甘草) |
| Luteolin | 5280445 | ACE2, 3CLpro, Spro, Rdrp | Gao LQ, et al. [76] Ye MB, et al. [79] Xing Y, et al. [90] Gao K, et al. [93] | Ephedra Herba (麻黄)，Forsythiae Fructus (连翘)，Lonicerae Japonicae Flos (金银花)，Menthae Haplocalycis Herba (薄荷)，Platycodon Grandiforus (桔梗)，Rhodiolae Crenulatae Radix et Rhizoma (红景天)，Codonopsis Radix (党参)，Polygoni Cuspidati Rhizoma et Radix (虎杖)，Artemisiae Annuae Herba (青蒿)，Salviae Miltiorrhizae Radix et Rhizoma (丹参)，Asteris Radix et Rhizoma (紫菀)，Belamcandae Rhizome (射干)，Verbenae Herb (马鞭草)，Herba Patriniae (败酱草)，Pseudostellariae Radix (太子参)，Aurantii Fructus Immaturus (枳实)，Eupatorii Herba (佩兰)，Carthami Flos (红花)，Taraxacl Herba (蒲公英)，Perillae Folium (紫苏叶) |
| Mairin | 64971 | ACE2, 3CLpro, Spro | Li XL, et al. [80] | Glycyrrhizae Radix et Rhizoma (甘草)，Armeniacae Semen Amarum (苦杏仁)，Forsythiae Fructus (连翘)，Astragali Radix (黄芪)，Paeoniae Radix Alba (白芍)，Jujubae Fructus (大枣)，Mori Cortex (桑白皮) |
| Mandenol | 5282184 | ACE2, 3CLpro, Spro | Li XL, et al. [80] | Ephedra Herba (麻黄)，Lonicerae Japonicae Flos (金银花)，Coicis Semen (薏苡仁)，Trichosanthis Fructus (瓜蒌)，Chuanxiong Rhizoma (川芎)，Angelicae Dahuricae Radix (白芷)，Gardeniae Fructus (栀子) |
| Mangiferin | 5281647 | ACE2 | Ren X, et al. [89] | Anemarrhenae Rhizoma (知母)，Belamcandae Rhizome (射干) |
| Medicarpin | 336327 | ACE2, 3CLpro, Spro | Li XL, et al. [80] | Glycyrrhizae Radix et Rhizoma (甘草) |
| Moslosooflavone | 188316 | 3CLpro, Rdrp | Ye MB, et al. [79] | Scutellariae Radix (黄芩)，Andrographis Herba (穿心莲) |
| N-[2-[(2R,3S)-3-acetamido-2-(3,4-dihydroxyphenyl)-2,3-dihydro-1,4-benzodioxin-6-yl] ethyl] acetamide | 10715163 | ACE2 | Ren X, et al. [89] | Cicadae Periostracum (蝉蜕) |
| Naringenin | 932 | ACE2, 3CLpro, Plpro | Tao QY, et al. [72] Chen J, et al. [74] | Glycyrrhizae Radix et Rhizoma (甘草)，Ephedra Herba (麻黄)，Citri Reticulatae Pericarpium (陈皮)，Menthae Haplocalycis Herba (薄荷)，Aurantii Fructus Immaturus (枳实) |
| Odoratin | 13965473 | ACE2, 3CLpro, Spro | Li XL, et al. [80] | Glycyrrhizae Radix et Rhizoma (甘草) |
| Oroxylin a | 5320315 | 3CLpro, Rdrp | Ye MB, et al. [79] | Scutellariae Radix (黄芩)，Andrographis Herba (穿心莲) |
| Pectolinarigenin | 5320438 | ACE2, 3CLpro, Spro | Li XL, et al. [80] | Ephedra Herba (麻黄) |
| Phaseol | 44257530 | ACE2, 3CLpro, Spro | Li XL, et al. [80] Gao K, et al. [93] | Glycyrrhizae Radix et Rhizoma (甘草)，Armeniacae Semen Amarum (苦杏仁) |
| Phaseolinisoflavan | 4484952 | ACE2, 3CLpro, Spro | Li XL, et al. [80] | Glycyrrhizae Radix et Rhizoma (甘草) |
| Physciondiglucoside | 73981703 | 3CLpro | Ren X, et al. [89] | Rhei Radix et Rhizoma (大黄），Polygoni Cuspidati Rhizoma et Radix (虎杖) |
| Poriferast-5-en-3beta-ol | 457801 | 3CLpro, Spro | Li XL, et al. [80] | Ephedra Herba (麻黄)，Isatidis Radix (板蓝根)，Zingiber Officinale Roscoe (生姜)，Salviae Miltiorrhizae Radix et Rhizoma (丹参)，Isatidis Folium (大青叶)，Amomi Fructus (砂仁)，Perillae Folium (紫苏叶)，Carthami Flos (红花)，Mori Follum (桑叶) |
| Procyanidin B1 | 11250133 | ACE2, 3CLpro | Wang J, et al. [84] | Arecae Semen (槟榔) |
| Puerarin | 5281807 | ACE2, 3CLpro, Spro | Gao LQ, et al. [76] Pan BY, et al. [92] | Bupleuri Radix (柴胡) |
| Quercetin | 5280343 | ACE2, 3CLpro, Spro, Rdrp | Tao QY, et al. [72] Gao LQ, et al. [76] Ye MB, et al. [79] Ren X, et al. [89] Xing Y, et al. [90] Gao K, et al. [93] | Glycyrrhizae Radix et Rhizoma (甘草)，Ephedra Herba (麻黄)，Forsythiae Fructus (连翘)，Pogostemonis Herba (广藿香)，Lonicerae Japonicae Flos (金银花)，Bupleuri Radix (柴胡)，Tsaoko Fructus (草果)，Houttuyniae Herba (鱼腥草)，Astragali Radix (黄芪)，Rhodiolae Crenulatae Radix et Rhizoma (红景天)，Descurainiae Semen (葶苈子)，Polygoni Cuspidati Rhizoma et Radix (虎杖)，Artemisiae Annuae Herba (青蒿)，Farfarae Flos (款冬花)，Asteris Radix et Rhizoma (紫菀)，Peucedani Radix (前胡)，Verbenae Herb (马鞭草)，Herba Patriniae (败酱草)，Mume Fructus (乌梅)，Carthami Flos (红花)，Taraxacl Herba (蒲公英)，Crataegi Fructs (山楂)，Coptidis Rhizoma (黄连)，Artemisiae Scopariae Herba (茵陈)，Gardeniae Fructus (栀子)，Mori Cortex (桑白皮) |
| Quercetin 3,3'-dimethyl ether | 5316900 | ACE2, 3CLpro, Spro | Li XL, et al. [80] | Glycyrrhizae Radix et Rhizoma (甘草) |
| Quercimeritrin | 5282160 | ACE2 | Ren X, et al. [89] | Pogostemonis Herba (广藿香) |
| Resivit | 440833 | ACE2, 3CLpro, Spro | Li XL, et al. [80] | Ephedra Herba (麻黄)，Arecae Semen (槟榔) |
| Rhein | 10168 | Nsp14 | Cai Y, et al. [83] | Rhei Radix et Rhizoma (大黄），Polygoni Cuspidati Rhizoma et Radix (虎杖) |
| Rivularin | 13889022 | 3CLpro, Rdrp | Ye MB, et al. [79] | Scutellariae Radix (黄芩) |
| Rosmarinic acid | 5281792 | ACE2, 3CLpro | Wang H, et al. [82] | Salviae Miltiorrhizae Radix et Rhizoma (丹参) |
| Rutin | 5280805 | ACE2, 3CLpro, Spro, Rdrp | Ye MB, et al. [79] Wu HY, et al. [81] Xing Y, et al. [90] | Glycyrrhizae Radix et Rhizoma (甘草)，Ephedra Herba (麻黄)，Forsythiae Fructus (连翘)，Lonicerae Japonicae Flos (金银花)，Bupleuri Radix (柴胡)，Houttuyniae Herba (鱼腥草)，Astragali Radix (黄芪)，Rhodiolae Crenulatae Radix et Rhizoma (红景天)，Farfarae Flos (款冬花)，Asteris Radix et Rhizoma (紫菀)，Herba Patriniae (败酱草)，Carthami Flos (红花)，Taraxacl Herba (蒲公英)，Crataegi Fructs (山楂)，Mori Follum (桑叶)，Artemisiae Scopariae Herba (茵陈)，Gardeniae Fructus (栀子)，Mori Cortex (桑白皮) |
| Salvianolic acid B | 11629084 | ACE2, 3CLpro, Spro | Xing Y, et al. [90] | Salviae Miltiorrhizae Radix et Rhizoma (丹参) |
| Salvigenin | 161271 | ACE2, 3CLpro, Spro, Rdrp | Ye MB, et al. [79] | Scutellariae Radix (黄芩) |
| Semilicoisoflavone B | 5481948 | ACE2, 3CLpro, Spro | Li XL, et al. [80] | Glycyrrhizae Radix et Rhizoma (甘草) |
| Sennoside D | 46173830 | 3CLpro | Ren X, et al. [79] | Radix Rhei Et Rhizome (大黄) |
| Shinpterocarpin | 10336244 | ACE2, 3CLpro, Spro | Li XL, et al. [80] | Glycyrrhizae Radix et Rhizoma (甘草) |
| Sigmoidin-B | 73205 | ACE2, 3CLpro, Spro | Li XL, et al. [80] | Glycyrrhizae Radix et Rhizoma (甘草) |
| Sitosterol | 12303645 | ACE2, 3CLpro, Spro, Rdrp | Tao QY, et al. [72] Li XL, et al. [78] Ye MB, et al. [79] Gao K, et al. [93] | Glycyrrhizae Radix et Rhizoma (甘草)，Armeniacae Semen Amarum (苦杏仁)，Scutellariae Radix (黄芩)，Citri Reticulatae Pericarpium (陈皮)，Isatidis Radix (板蓝根)，Menthae Haplocalycis Herba (薄荷)，Artemisiae Annuae Herba (青蒿)，Cinnamomi Ramulus (桂枝)，Paeoniae Radix Rubra (赤芍)，Scrophulariae Radix (玄参)，Coicis Semen (薏苡仁)，Peucedani Radix (前胡)，Fritiliariae Cirrhosae Bulbus (川贝母)，Alismatis Rhizoma (泽泻)，Aconiti Lateralis Radix Praeparata (附子)，Herba Patriniae (败酱草)，Hordei Fructus Germinatus (麦芽)，Notopterygii Rhizoma et Radix (羌活)，Eupatorii Herba (佩兰)，Chuanxiong Rhizoma (川芎)，Zingiberis Rhizoma (干姜)，Cynanchi Paniculati Radix et Rhizoma (徐长卿)，Tetrapanacis Medulla (通草)，Paeoniae Radix Alba (白芍)，Sargentodoxae Caulis (大血藤) |
| Stigmasterol | 5280794 | ACE2, 3CLpro, Spro, Rdrp | Tao QY, et al. [72] Li XL, et al. [78] Ye MB, et al. [79] Gao K, et al. [93] | Armeniacae Semen Amarum (苦杏仁)，Ephedra Herba (麻黄)，Scutellariae Radix (黄芩)，Pinelliae Rhizoma Praeparatum (法半夏)，Lonicerae Japonicae Flos (金银花)，Bupleuri Radix (柴胡)，Isatidis Radix (板蓝根)，Zingiber Officinale Roscoe (生姜)，Codonopsis Radix (党参)，Artemisiae Annuae Herba (青蒿)，Ginseng Radix et Rhizoma (人参)，Anemarrhenae Rhizoma (知母)，Paeoniae Radix Rubra (赤芍)，Belamcandae Rhizome (射干)，Rhizoma Dioscoreae (山药)，Coicis Semen (薏苡仁)，Verbenae Herb (马鞭草)，Ophiopogon japonicus (麦冬)，Herba Patriniae (败酱草)，Angelicae Sinensis Radix (当归)，Amomi Fructus (砂仁)，Cremastrae Pseudobulbus (山慈菇)，Mume Fructus (乌梅)，Eupatorii Herba (佩兰)，Carthami Flos (红花)，Curcumaelongae Rhizoma (姜黄)，Lilii Bulbus (百合)，Mori Follum (桑叶)，Angelicae Dahuricae Radix (白芷)，Gardeniae Fructus (栀子)，Jujubae Fructus (大枣) |
| Supraene | 638072 | ACE2, 3CLpro | Li XL, et al. [80] | Ephedra Herba (麻黄)，Scutellariae Radix (黄芩)，Pseudostellariae Radix (太子参)，Arctii Fructus (牛蒡子)，Perillae Folium (紫苏叶)，Mori Follum (桑叶)，Angelicae Dahuricae Radix (白芷)，Gardeniae Fructus (栀子) |
| Tanshinone iia | 164676 | 3CLpro, Rdrp | Ye MB, et al. [79] | Salviae Miltiorrhizae Radix et Rhizoma (丹参)，Peucedani Radix (前胡) |
| Taxifolin | 439533 | ACE2, 3CLpro, Spro | Li XL, et al. [80] | Ephedra Herba (麻黄)，Cinnamomi Ramulus (桂枝) |
| Truflex OBP | 66540 | ACE2, 3CLpro, Spro | Li XL, et al. [80] | Ephedra Herba (麻黄) |
| Vestitol | 92503 | ACE2, 3CLpro, Spro | Li XL, et al. [80] | Glycyrrhizae Radix et Rhizoma (甘草) |
| Wogonin | 5281703 | 3CLpro, Rdrp | Ye MB, et al. [79] | Forsythiae Fructus (连翘)，Scutellariae Radix (黄芩)，Atractylodis Rhizoma (苍术)，Andrographis Herba (穿心莲) |
| Xambioona | 14769500 | ACE2, 3CLpro, Spro | Li XL, et al. [80] | Glycyrrhizae Radix et Rhizoma (甘草) |

**References**

1. Tao Q, Du J, Li X, Zeng J, Tan B, Xu J, et al. Network pharmacology and molecular docking analysis on molecular targets and mechanisms of Huashi Baidu formula in the treatment of COVID-19. *Drug Dev Ind Pharm.* 2020;**46**:1345-53.
2. Xia QD, Xun Y, Lu JL, Lu YC, Yang YY, Zhou P, et al. Network pharmacology and molecular docking analyses on Lianhua Qingwen capsule indicate Akt1 is a potential target to treat and prevent COVID-19. *Cell Prolif.* 2020;**53**:e12949.
3. Chen J, Wang YK, Gao Y, Hu LS, Yang JW, Wang JR, et al. Protection against COVID-19 injury by qingfei paidu decoction via anti-viral, anti-inflammatory activity and metabolic programming. *Biomed Pharmacother.* 2020;**129**:110281.
4. Mu C, Sheng Y, Wang Q, Amin A, Li X, and Xie Y. Potential compound from herbal food of Rhizoma Polygonati for treatment of COVID-19 analyzed by network pharmacology: Viral and cancer signaling mechanisms. *J Funct Foods.* 2021;**77**:104149.
5. Gao LQ, Xu J, and Chen SD. In Silico Screening of Potential Chinese Herbal Medicine Against COVID-19 by Targeting SARS-CoV-2 3CLpro and Angiotensin Converting Enzyme II Using Molecular Docking. *Chin J Integr Med.* 2020;**26**:527-32.
6. Ge C, and He Y. In Silico Prediction of Molecular Targets of Astragaloside IV for Alleviation of COVID-19 Hyperinflammation by Systems Network Pharmacology and Bioinformatic Gene Expression Analysis. *Front Pharmacol.* 2020;**11**:556984.
7. Li X, Lin H, Wang Q, Cui L, Luo H, and Luo L. Chemical composition and pharmacological mechanism of shenfu decoction in the treatment of novel coronavirus pneumonia (COVID-19). *Drug Dev Ind Pharm.* 2020;**46**:1947-59.
8. Ye M, Luo G, Ye D, She M, Sun N, Lu YJ, et al. Network pharmacology, molecular docking integrated surface plasmon resonance technology reveals the mechanism of Toujie Quwen Granules against coronavirus disease 2019 pneumonia. *Phytomedicine.* 2021;**85**:153401.
9. Li X, Qiu Q, Li M, Lin H, Cao S, Wang Q, et al. Chemical composition and pharmacological mechanism of ephedra-glycyrrhiza drug pair against coronavirus disease 2019 (COVID-19). *Aging (Albany NY).* 2021;**13**:4811-30.
10. Wu H, Gong K, Qin Y, Yuan Z, Xia S, Zhang S, et al. In silico analysis of the potential mechanism of a preventive Chinese medicine formula on coronavirus disease 2019. *J Ethnopharmacol.* 2021;**275**:114098.
11. Wang H, Zhang J, Lu Z, Dai W, Ma C, Xiang Y, et al. Identification of potential therapeutic targets and mechanisms of COVID-19 through network analysis and screening of chemicals and herbal ingredients. *Brief Bioinform.* 2021.
12. Cai Y, Zeng M, and Chen YZ. The pharmacological mechanism of Huashi Baidu Formula for the treatment of COVID-19 by combined network pharmacology and molecular docking. *Ann Palliat Med.* 2021;**10**:3864-95.
13. Wang J, Ge W, Peng X, Yuan L, He S, and Fu X. Investigating the active compounds and mechanism of HuaShi XuanFei formula for prevention and treatment of COVID-19 based on network pharmacology and molecular docking analysis. *Mol Divers.* 2021.
14. Xiao Z, Ye Q, Duan X, and Xiang T. Network Pharmacology Reveals That Resveratrol Can Alleviate COVID-19-Related Hyperinflammation. *Dis Markers.* 2021;**2021**:4129993.
15. Li Y, Chu F, Li P, Johnson N, Li T, Wang Y, et al. Potential effect of Maxing Shigan decoction against coronavirus disease 2019 (COVID-19) revealed by network pharmacology and experimental verification. *J Ethnopharmaco.l* 2021;**271**:113854.
16. Du HX, Zhu JQ, Chen J, Zhou HF, Yang JH, and Wan HT. Revealing the therapeutic targets and molecular mechanisms of emodin-treated coronavirus disease 2019 via a systematic study of network pharmacology. *Aging (Albany NY).* 2021;**13**:14571-89.
17. Niu WH, Wu F, Cao WY, Wu ZG, Chao YC, and Liang C. Network pharmacology for the identification of phytochemicals in traditional Chinese medicine for COVID-19 that may regulate interleukin-6. *Biosci Rep.* 2021;**41**.
18. Ren X, Shao XX, Li XX, Jia XH, Song T, Zhou WY, et al. Identifying potential treatments of COVID-19 from Traditional Chinese Medicine (TCM) by using a data-driven approach. *J Ethnopharmacol.* 2020;**258**:112932.
19. Xing Y, Hua YR, Shang J, Ge WH, and Liao J. Traditional Chinese medicine network pharmacology study on exploring the mechanism of Xuebijing Injection in the treatment of coronavirus disease 2019. *Chin J Nat Med.* 2020;**18**:941-51.
20. Ruan X, Du P, Zhao K, Huang J, Xia H, Dai D, et al. Mechanism of Dayuanyin in the treatment of coronavirus disease 2019 based on network pharmacology and molecular docking. *Chin Med.* 2020;**15**:62.
21. Pan B, Fang S, Zhang J, Pan Y, Liu H, Wang Y, et al. Chinese herbal compounds against SARS-CoV-2: Puerarin and quercetin impair the binding of viral S-protein to ACE2 receptor. *Comput Struct Biotechnol J.* 2020;**18**:3518-27.
22. Gao K, Song YP, and Song A. Exploring active ingredients and function mechanisms of Ephedra-bitter almond for prevention and treatment of Corona virus disease 2019 (COVID-19) based on network pharmacology. *BioData Min.* 2020;**13**:19.
23. Yu MX, Song X, Ma XQ, Hao CX, Huang JJ, and Yang WH. Investigation into molecular mechanisms and high-frequency core TCM for pulmonary fibrosis secondary to COVID-19 based on network pharmacology and data mining. *Ann Palliat Med.* 2021;**10**:3960-75.
