# Supplementary material for: Efficacy and mechanisms of traditional Chinese medicine for COVID-19: a systematic review
Source: Chin Med. 2022 Feb 28;17:30. doi: 10.1186/s13020-022-00587-7 (PMC8883015; doi:10.1186/s13020-022-00587-7)
Supplement: Supplementary file 8 — Additional file 8. Anti-inflammatory or immune-regulating components and targets. [file 13020_2022_587_MOESM8_ESM.docx]

Additional file 8. Anti-inflammatory or immune-regulating components and targets

| Component | PubChem CID | Targets | References | Chinese medicinal herbs |
| --- | --- | --- | --- | --- |
| 7-Methoxy-2-methyl isoflavone | 354368 | IL-6, IL1B, CCL2 | Ruan XF, et al. [91] | Glycyrrhizae Radix et Rhizoma (甘草)，Codonopsis Radix (党参) |
| Apigenin | 5280443 | IL-6, TNF | Wang H, et al. [82] | Ephedra Herba (麻黄)，Scutellariae Radix (黄芩)，Pogostemonis Herba (广藿香)，Lonicerae Japonicae Flos (金银花)，Isatidis Radix (板蓝根)，Menthae Haplocalycis Herba (薄荷)，Codonopsis Radix (党参)，Polygoni Cuspidati Rhizoma et Radix (虎杖)，Artemisiae Annuae Herba (青蒿)，Salviae Miltiorrhizae Radix et Rhizoma (丹参)，Asteris Radix et Rhizoma (紫菀)，Belamcandae Rhizome (射干)，Verbenae Herb (马鞭草)，Herba Patriniae (败酱草)，Aurantii Fructus Immaturus (枳实)，Carthami Flos (红花)，Perillae Folium (紫苏叶)，Acori Tataninowii Rhizoma (石菖蒲） |
| Artemetin | 5320351 | IL-6 | Ye MB, et al. [79] | Artemisiae Annuae Herba (青蒿)，Verbenae Herb (马鞭草) |
| Astragaloside IV | 13943297 | IL-6 | Ge CL, et al. [77] Ye MB, et al. [79] | Astragali Radix (黄芪) |
| Atractylenolide I | 5321018 | IL-6 | Niu WH, et al. [88] | Atractylodis Macrocephalae Rhizoma (白术)，Atractylodis Rhizoma (苍术) |
| Baicalein | 5281605 | IL-6 | Ye MB, et al. [79] | Scutellariae Radix (黄芩)，Pinelliae Rhizoma Praeparatum (法半夏)，Paeoniae Radix Rubra (赤芍)，Carthami Flos (红花) |
| Beta-carotene | 5280489 | IL-6, AKT1 | Xia QD, et al. [73] Ye MB, et al. [79] | Carthami Flos (红花)，Lablab Semen Album (白扁豆)，Jujubae Fructus (大枣) |
| Bicuculline | 10237 | IL-6 | Ye MB, et al. [79] | Forsythiae Fructus (连翘) |
| Calycosin | 5280448 | IL-6 | Ye MB, et al. [79] | Glycyrrhizae Radix et Rhizoma (甘草)，Astragali Radix (黄芪) |
| Emodin | 3220 | TNF, IL1B | Du HX, et al. [87] | Rhei Radix et Rhizoma (大黄），Isatidis Radix (板蓝根)，Menthae Haplocalycis Herba (薄荷)，Lilii Bulbus (百合)，Sargentodoxae Caulis (大血藤) |
| Formononetin | 5280378 | IL-6, IL1B, CCL2 | Ruan XF, et al. [91] | Glycyrrhizae Radix et Rhizoma (甘草)，Astragali Radix (黄芪) |
| Indirubin | 10177 | IL-6 | Ye MB, et al. [79] | Isatidis Folium (大青叶) |
| Isoquercitrin | 5280804 | IL-6 | Ye MB, et al. [79] | Bupleuri Radix (柴胡)，Tsaoko Fructus (草果)，Herba Patriniae (败酱草) |
| kaempferol | 5280863 | IL-6, TNF, IL1B, CCL2, AKT1 | Xia QD, et al. [73] Ye MB, et al. [79] Wang H, et al. [82] Ruan XF, et al. [91] | Glycyrrhizae Radix et Rhizoma (甘草)，Ephedra Herba (麻黄)，Forsythiae Fructus (连翘)，Lonicerae Japonicae Flos (金银花)，Bupleuri Radix (柴胡)，Dryopteridis Crassirhizomatis Rhizoma (绵马贯众)，Houttuyniae Herba (鱼腥草)，Astragali Radix (黄芪)，Rhodiolae Crenulatae Radix et Rhizoma (红景天)，Descurainiae Semen (葶苈子)，Artemisiae Annuae Herba (青蒿)，Ginseng Radix et Rhizoma (人参)，Anemarrhenae Rhizoma (知母)，Farfarae Flos (款冬花)，Asteris Radix et Rhizoma (紫菀)，Verbenae Herb (马鞭草)，Herba Patriniae (败酱草)，Fructus Arctii (牛蒡子)，Asari Radix et Rhizoma (细辛)，Mume Fructus (乌梅)，Carthami Flos (红花)，Mori Follum (桑叶)，Paeoniae Radix Alba (白芍)，Gardeniae Fructus (栀子)，Mori Cortex (桑白皮) |
| Luteolin | 5280445 | IL-6, AKT1 | Xia QD, et al. [73] Niu WH, et al. [88] | Ephedra Herba (麻黄)，Forsythiae Fructus (连翘)，Lonicerae Japonicae Flos (金银花)，Menthae Haplocalycis Herba (薄荷)，Platycodon Grandiforus (桔梗)，Rhodiolae Crenulatae Radix et Rhizoma (红景天)，Codonopsis Radix (党参)，Polygoni Cuspidati Rhizoma et Radix (虎杖)，Artemisiae Annuae Herba (青蒿)，Salviae Miltiorrhizae Radix et Rhizoma (丹参)，Asteris Radix et Rhizoma (紫菀)，Belamcandae Rhizome (射干)，Verbenae Herb (马鞭草)，Herba Patriniae (败酱草)，Pseudostellariae Radix (太子参)，Aurantii Fructus Immaturus (枳实)，Eupatorii Herba (佩兰)，Carthami Flos (红花)，Taraxacl Herba (蒲公英)，Perillae Folium (紫苏叶) |
| Myricetin | 5281672 | IL-6 | Niu WH, et al. [88] | Forsythiae Fructus (连翘)，Carthami Flos (红花) |
| Naringenin | 932 | IL-6, IL1B, CCL2, AKT1 | Xia QD, et al. [73] Ruan XF, et al. [91] | Glycyrrhizae Radix et Rhizoma (甘草)，Ephedra Herba (麻黄)，Citri Reticulatae Pericarpium (陈皮)，Menthae Haplocalycis Herba (薄荷)，Aurantii Fructus Immaturus (枳实) |
| Paeoniflorin | 442534 | IL-6 | Niu WH, et al. [88] | Paeoniae Radix Rubra (赤芍))，Paeoniae Radix Alba (白芍) |
| Quercetin | 5280343 | IL-6, TNF, IL1B, CCL2, AKT1 | Xia QD, et al. [73] Ye MB, et al. [79] Niu WH, et al. [88] Ruan XF, et al. [91] Yu MX, et al. [94] | Glycyrrhizae Radix et Rhizoma (甘草)，Ephedra Herba (麻黄)，Forsythiae Fructus (连翘)，Pogostemonis Herba (广藿香)，Lonicerae Japonicae Flos (金银花)，Bupleuri Radix (柴胡)，Tsaoko Fructus (草果)，Houttuyniae Herba (鱼腥草)，Astragali Radix (黄芪)，Rhodiolae Crenulatae Radix et Rhizoma (红景天)，Descurainiae Semen (葶苈子)，Polygoni Cuspidati Rhizoma et Radix (虎杖)，Artemisiae Annuae Herba (青蒿)，Farfarae Flos (款冬花)，Asteris Radix et Rhizoma (紫菀)，Peucedani Radix (前胡)，Verbenae Herb (马鞭草)，Herba Patriniae (败酱草)，Mume Fructus (乌梅)，Carthami Flos (红花)，Taraxacl Herba (蒲公英)，Crataegi Fructs (山楂)，Coptidis Rhizoma (黄连)，Artemisiae Scopariae Herba (茵陈)，Gardeniae Fructus (栀子)，Mori Cortex (桑白皮) |
| Resveratrol | 445154 | IL-6, TNF, IL1B | Niu WH, et al. [88] Yu MX, et al. [94] | Polygoni Cuspidati Rhizoma et Radix (虎杖)，Mori Cortex (桑白皮) |
| Rosmarinic acid | 5281792 | IL-6, TNF | Wang H, et al. [82] | Salviae Miltiorrhizae Radix et Rhizoma (丹参) |
| Rutin | 5280805 | IL-6 | Ye MB, et al. [79] Niu WH, et al. [88] | Glycyrrhizae Radix et Rhizoma (甘草)，Ephedra Herba (麻黄)，Forsythiae Fructus (连翘)，Lonicerae Japonicae Flos (金银花)，Bupleuri Radix (柴胡)，Houttuyniae Herba (鱼腥草)，Astragali Radix (黄芪)，Rhodiolae Crenulatae Radix et Rhizoma (红景天)，Farfarae Flos (款冬花)，Asteris Radix et Rhizoma (紫菀)，Herba Patriniae (败酱草)，Carthami Flos (红花)，Taraxacl Herba (蒲公英)，Crataegi Fructs (山楂)，Mori Follum (桑叶)，Artemisiae Scopariae Herba (茵陈)，Gardeniae Fructus (栀子)，Mori Cortex (桑白皮) |
| Salvigenin | 161271 | IL-6 | Ye MB, et al. [79] | Scutellariae Radix (黄芩) |
| Ursolic acid | 64945 | IL-6 | Niu WH, et al. [88] | Glycyrrhizae Radix et Rhizoma (甘草)，Armeniacae Semen Amarum (苦杏仁)，Ephedra Herba (麻黄)，Forsythiae Fructus (连翘)，Lonicerae Japonicae Flos (金银花)，Menthae Haplocalycis Herba (薄荷)，Salviae Miltiorrhizae Radix et Rhizoma (丹参)，Scrophulariae Radix (玄参)，Peucedani Radix (前胡)，Verbenae Herb (马鞭草)，Herba Patriniae (败酱草)，Pseudostellariae Radix (太子参)，Mume Fructus (乌梅)，Crataegi Fructs (山楂)，Perillae Folium (紫苏叶)，Mori Follum (桑叶)，Gardeniae Fructus (栀子)，Mori Cortex (桑白皮) |
| Wogonin | 5281703 | IL-6, AKT1 | Xia QD, et al. [73] Niu WH, et al. [88] | Forsythiae Fructus (连翘)，Scutellariae Radix (黄芩)，Atractylodis Rhizoma (苍术)，Andrographis Herba (穿心莲) |

**References**

1. Tao Q, Du J, Li X, Zeng J, Tan B, Xu J, et al. Network pharmacology and molecular docking analysis on molecular targets and mechanisms of Huashi Baidu formula in the treatment of COVID-19. *Drug Dev Ind Pharm.* 2020;**46**:1345-53.
2. Xia QD, Xun Y, Lu JL, Lu YC, Yang YY, Zhou P, et al. Network pharmacology and molecular docking analyses on Lianhua Qingwen capsule indicate Akt1 is a potential target to treat and prevent COVID-19. *Cell Prolif.* 2020;**53**:e12949.
3. Chen J, Wang YK, Gao Y, Hu LS, Yang JW, Wang JR, et al. Protection against COVID-19 injury by qingfei paidu decoction via anti-viral, anti-inflammatory activity and metabolic programming. *Biomed Pharmacother.* 2020;**129**:110281.
4. Mu C, Sheng Y, Wang Q, Amin A, Li X, and Xie Y. Potential compound from herbal food of Rhizoma Polygonati for treatment of COVID-19 analyzed by network pharmacology: Viral and cancer signaling mechanisms. *J Funct Foods.* 2021;**77**:104149.
5. Gao LQ, Xu J, and Chen SD. In Silico Screening of Potential Chinese Herbal Medicine Against COVID-19 by Targeting SARS-CoV-2 3CLpro and Angiotensin Converting Enzyme II Using Molecular Docking. *Chin J Integr Med.* 2020;**26**:527-32.
6. Ge C, and He Y. In Silico Prediction of Molecular Targets of Astragaloside IV for Alleviation of COVID-19 Hyperinflammation by Systems Network Pharmacology and Bioinformatic Gene Expression Analysis. *Front Pharmacol.* 2020;**11**:556984.
7. Li X, Lin H, Wang Q, Cui L, Luo H, and Luo L. Chemical composition and pharmacological mechanism of shenfu decoction in the treatment of novel coronavirus pneumonia (COVID-19). *Drug Dev Ind Pharm.* 2020;**46**:1947-59.
8. Ye M, Luo G, Ye D, She M, Sun N, Lu YJ, et al. Network pharmacology, molecular docking integrated surface plasmon resonance technology reveals the mechanism of Toujie Quwen Granules against coronavirus disease 2019 pneumonia. *Phytomedicine.* 2021;**85**:153401.
9. Li X, Qiu Q, Li M, Lin H, Cao S, Wang Q, et al. Chemical composition and pharmacological mechanism of ephedra-glycyrrhiza drug pair against coronavirus disease 2019 (COVID-19). *Aging (Albany NY).* 2021;**13**:4811-30.
10. Wu H, Gong K, Qin Y, Yuan Z, Xia S, Zhang S, et al. In silico analysis of the potential mechanism of a preventive Chinese medicine formula on coronavirus disease 2019. *J Ethnopharmacol.* 2021;**275**:114098.
11. Wang H, Zhang J, Lu Z, Dai W, Ma C, Xiang Y, et al. Identification of potential therapeutic targets and mechanisms of COVID-19 through network analysis and screening of chemicals and herbal ingredients. *Brief Bioinform.* 2021.
12. Cai Y, Zeng M, and Chen YZ. The pharmacological mechanism of Huashi Baidu Formula for the treatment of COVID-19 by combined network pharmacology and molecular docking. *Ann Palliat Med.* 2021;**10**:3864-95.
13. Wang J, Ge W, Peng X, Yuan L, He S, and Fu X. Investigating the active compounds and mechanism of HuaShi XuanFei formula for prevention and treatment of COVID-19 based on network pharmacology and molecular docking analysis. *Mol Divers.* 2021.
14. Xiao Z, Ye Q, Duan X, and Xiang T. Network Pharmacology Reveals That Resveratrol Can Alleviate COVID-19-Related Hyperinflammation. *Dis Markers.* 2021;**2021**:4129993.
15. Li Y, Chu F, Li P, Johnson N, Li T, Wang Y, et al. Potential effect of Maxing Shigan decoction against coronavirus disease 2019 (COVID-19) revealed by network pharmacology and experimental verification. *J Ethnopharmaco.l* 2021;**271**:113854.
16. Du HX, Zhu JQ, Chen J, Zhou HF, Yang JH, and Wan HT. Revealing the therapeutic targets and molecular mechanisms of emodin-treated coronavirus disease 2019 via a systematic study of network pharmacology. *Aging (Albany NY).* 2021;**13**:14571-89.
17. Niu WH, Wu F, Cao WY, Wu ZG, Chao YC, and Liang C. Network pharmacology for the identification of phytochemicals in traditional Chinese medicine for COVID-19 that may regulate interleukin-6. *Biosci Rep.* 2021;**41**.
18. Ren X, Shao XX, Li XX, Jia XH, Song T, Zhou WY, et al. Identifying potential treatments of COVID-19 from Traditional Chinese Medicine (TCM) by using a data-driven approach. *J Ethnopharmacol.* 2020;**258**:112932.
19. Xing Y, Hua YR, Shang J, Ge WH, and Liao J. Traditional Chinese medicine network pharmacology study on exploring the mechanism of Xuebijing Injection in the treatment of coronavirus disease 2019. *Chin J Nat Med.* 2020;**18**:941-51.
20. Ruan X, Du P, Zhao K, Huang J, Xia H, Dai D, et al. Mechanism of Dayuanyin in the treatment of coronavirus disease 2019 based on network pharmacology and molecular docking. *Chin Med.* 2020;**15**:62.
21. Pan B, Fang S, Zhang J, Pan Y, Liu H, Wang Y, et al. Chinese herbal compounds against SARS-CoV-2: Puerarin and quercetin impair the binding of viral S-protein to ACE2 receptor. *Comput Struct Biotechnol J.* 2020;**18**:3518-27.
22. Gao K, Song YP, and Song A. Exploring active ingredients and function mechanisms of Ephedra-bitter almond for prevention and treatment of Corona virus disease 2019 (COVID-19) based on network pharmacology. *BioData Min.* 2020;**13**:19.
23. Yu MX, Song X, Ma XQ, Hao CX, Huang JJ, and Yang WH. Investigation into molecular mechanisms and high-frequency core TCM for pulmonary fibrosis secondary to COVID-19 based on network pharmacology and data mining. *Ann Palliat Med.* 2021;**10**:3960-75.
